# FleA Expression in *Aspergillus fumigatus* Is Recognized by Fucosylated Structures on Mucins and Macrophages to Prevent Lung Infection

**DOI:** 10.1371/journal.ppat.1005555

**Published:** 2016-04-08

**Authors:** Sheena C. Kerr, Gregory J. Fischer, Meenal Sinha, Orla McCabe, Jonathan M. Palmer, Tsokyi Choera, Fang Yun Lim, Michaela Wimmerova, Stephen D. Carrington, Shaopeng Yuan, Clifford A. Lowell, Stefan Oscarson, Nancy P. Keller, John V. Fahy

**Affiliations:** 1 Division of Pulmonary and Critical Care Medicine, University of California, San Francisco, San Francisco, California; 2 Department of Genetics, University of Wisconsin, Madison, Madison, Wisconsin; 3 Department of Laboratory Medicine, University of California, San Francisco, San Francisco, California; 4 Center for Synthesis and Chemical Biology, University College Dublin, Dublin, Ireland; 5 Department of Medical Microbiology and Immunology, University of Wisconsin, Madison, Madison, Wisconsin; 6 Faculty of Science and Central European Institute of Technology, Masaryk University, Brno, Czech Republic; 7 Veterinary Science Centre, School of Agriculture, Food Science and Veterinary Medicine, University College Dublin, Dublin, Ireland; McGill University, CANADA

## Abstract

The immune mechanisms that recognize inhaled *Aspergillus fumigatus* conidia to promote their elimination from the lungs are incompletely understood. FleA is a lectin expressed by *Aspergillus fumigatus* that has twelve binding sites for fucosylated structures that are abundant in the glycan coats of multiple plant and animal proteins. The role of FleA is unknown: it could bind fucose in decomposed plant matter to allow *Aspergillus fumigatus* to thrive in soil, or it may be a virulence factor that binds fucose in lung glycoproteins to cause *Aspergillus fumigatus* pneumonia. Our studies show that FleA protein and *Aspergillus fumigatus* conidia bind avidly to purified lung mucin glycoproteins in a fucose-dependent manner. In addition, FleA binds strongly to macrophage cell surface proteins, and macrophages bind and phagocytose *fleA-*deficient *(∆fleA*) conidia much less efficiently than wild type (WT) conidia. Furthermore, a potent fucopyranoside glycomimetic inhibitor of FleA inhibits binding and phagocytosis of WT conidia by macrophages, confirming the specific role of fucose binding in macrophage recognition of WT conidia. Finally, mice infected with *ΔfleA* conidia had more severe pneumonia and invasive aspergillosis than mice infected with WT conidia. These findings demonstrate that FleA is not a virulence factor for *Aspergillus fumigatus*. Instead, host recognition of FleA is a critical step in mechanisms of mucin binding, mucociliary clearance, and macrophage killing that prevent *Aspergillus fumigatus* pneumonia.

## Author Summary

Inhaled *Aspergillus fumigatus* conidia are effectively eliminated from the lung by the coordinated actions of mucociliary clearance and macrophage killing, but the mechanisms of attachment of *Aspergillus fumigatus* (*A*. *fumigatus)* conidia to the airway mucus gel are unknown. In addition, the mechanisms of phagocytosis of conidia by macrophages are incompletely understood, because inhibition of Dectin-1, mannose receptor, and TLR-2/4 does not completely prevent phagocytosis. A fucose-binding lectin (FleA) expressed on the surface of *Aspergillus* conidia has recently been described, but its function is unknown. In order to reveal FleA’s function, we carried out combined *in vitro* and *in vivo* studies using several novel reagents, including recombinant FleA, FleA deficient *(∆fleA*) conidia and a potent fucopyranoside inhibitor of FleA. *In vitro* studies found that FleA mediates binding of *A*. *fumigatus* conidia to airway mucins and phagocytosis of conidia by lung macrophages. In *in vivo* studies we found that mice infected with *∆fleA* conidia develop invasive aspergillosis whereas those exposed to WT conidia do not. Based on our findings, we propose a novel host defense mechanism against *A*. *fumigatus* in which FleA expression on conidia is recognized by lung mucins and macrophages to promote mucociliary clearance and macrophage killing and protect from invasive pulmonary aspergillosis.

## Introduction

*Aspergillus fumigatus* (*A*. *fumigatus*) is an ubiquitous opportunistic pathogen that causes invasive and often fatal lung infection, particularly in immunocompromised patients [1]. *Aspergillus fumigatus* produces small hydrophobic conidia that are easily inhaled into the lungs and require robust host defense mechanisms to prevent infection. The mechanisms of clearance of conidia from the lung are incompletely understood but phagocytosis by macrophages is known to be important [2–5]. Macrophages express Dectin-1, a C-type lectin that recognizes β-1-3 glucan on the surface of *A*. *fumigatus* conidia. Although the amount of surface accessible β-1-3 glucan is low on resting conidia, it is much higher in swollen conidia that appear early during germination and infection [6]. Binding of β-glucan by Dectin-1 promotes macrophage killing of *A*. *fumigatus* conidia, and other macrophage receptors, such as the mannose receptor and toll-like receptors (TLR) -2 and -4, cooperate in this killing effect [7, 8]. Notably, however, the phagocytosis of *A*. *fumigatus* conidia by macrophages is incompletely blocked by inhibitors of Dectin-1, mannose receptor, and TLR-2/4 [9], which means that macrophages must employ additional mechanisms to phagocytose and kill *A*. *fumigatus*.

Many microorganisms use lectins as adhesins to interact with host glycoproteins. For example, *Pseudomonas*, *Burkholderia*, *Ralstonia*, and *Chromobacterium* bacteria all express adhesins that include galactophilic and fucophilic lectins that initiate bacterial adherence to glycan receptors on host cells and tissues [10–14]. In addition, fungi such as *Aleuria aurantia*, *A*. *oryzae*, and *A*. *fumigatus* all express fucophilic lectins [15–18] with multiple fucose binding sites [11, 19]. For example, *A*. *fumigatus* lectin (AFL, also known as FleA) exists as a dimer with 12 fucose-binding sites available for strong multivalent interactions with fucosylated structures [18]. As a result, FleA has unusually high binding affinity for fucosylated structures [18, 20], but its role in fungal biology is unknown. Fucosylated glycans are abundant in plants and animals, and FleA expression by fungi may help them bind plant or animal tissues, as has been found for fucose binding lectins in bacteria [11, 21]. *Aspergillus fumigatus* conidia enter the human host via inhalation, and fucosylated proteins in the lung that could bind conidial FleA include mucins in the airway mucus gel and multiple glycoproteins in the glycocalyx of macrophages [22–24]. Fucose in different linkages is a common carbohydrate structure in gel-forming mucins [22] such as MUC5AC and MUC5B [23], and recent studies in transgenic mice have revealed the essential role of gel-forming mucins in host defense against lung infection [25]. In addition, membrane-tethered mucins (such as MUC1 and MUC4) function as receptors in epithelial cells [26] and macrophages [24] and it is possible that they may function as adhesins for *A*. *fumigatus*. To determine the role of FleA in the pathogenesis of *A*. *fumigatus* pneumonia, we studied the behavior of recombinant FleA and *fleA-*deficient conidia in multiple complimentary functional assays, including mucin binding assays, macrophage binding assays, and a mouse model of *A*. *fumigatus* pneumonia.

## Results

### Phylogenetic analysis of FleA

We generated a phylogenetic tree to illustrate the fungal genera and species that contain a putative FleA ortholog. The tree shows that FleA is not widespread in the Kingdom Fungi and is found in several pathogenic species of fungi. FleA is present in the genomes of only a few *Aspergillus* species, including *A*. *fumigatus*, *A*. *flavus*, *A*. *oryzae and A*. *calidoustus* (S1 Fig). FleA is also present in other human pathogens including dermatophytes (*Trichophyton*, *Microsporum* and *Arthroderma)*, entomopathogenic fungi (*Metarhizium*, *Ophiocordyceps)*, a nematode pathogen (*Arthrobotrys oligospora*), several plant pathogens (*Penicillium expansum*, *Marssonina*, *Magnaporthiopsis*, *Ceratocystis*, *Gaumannomyces*, *Rhizoctonia*) and *Trichoderma* spp. (pathogenic on other fungi).

### FleA mediates binding of *A*. *fumigatus* conidia to airway mucin in a fucose dependent manner

**Fig 1:**
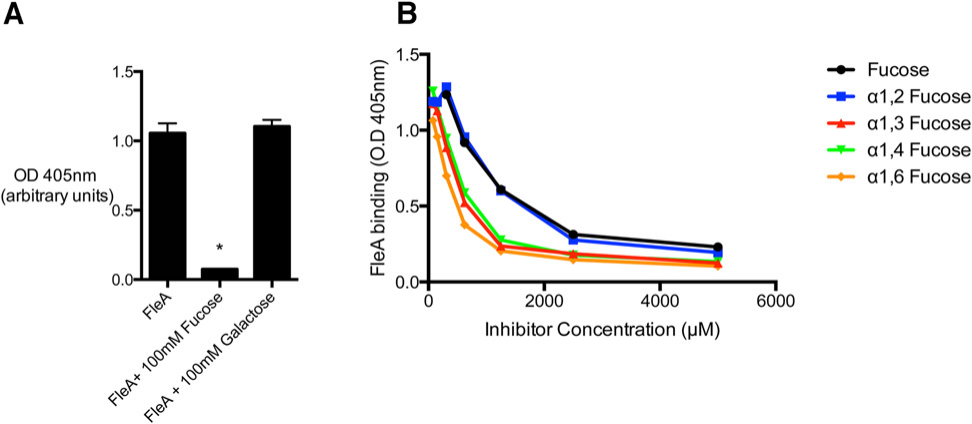
FleA binding to airway mucin is inhibited by fucose. (A) Biotinylated FleA binding to mucin is inhibited by fucose, but not by galactose. The substrate is mucin purified separately from induced sputum collected from 5 donors and then pooled. Data shown is a representative experiment +/- S.D of 3 replicates, representative of at least 3 experiments. *Denotes significantly different from control, p = <0.0001. (B) α1,2, α1,3, α1,4 and α1,6 linked fucose all inhibit FleA binding to mucin in a dose dependent manner.

To determine if *A*. *fumigatus* conidia can bind to mucin glycans in a FleA-dependent manner, we coated microtiter plates with mucins that we purified from the induced sputum of healthy subjects, and we used labeled recombinant FleA to quantify FleA-mucin binding. We found that FleA binds strongly to airway mucin and that this binding is inhibited by fucose (Fig 1A). No inhibition was observed with galactose (Fig 1A). To examine the specificity of FleA for the different linkage forms of fucose found naturally on mucins, we synthesized disaccharides with fucose linked to glucose in α1,2, α1,3, α1,4, or α1,6 positions. These fucose-glucose compounds all strongly inhibited FleA binding to mucin in a dose dependent manner (Fig 1B).

### Generation of *ΔfleA A*. *fumigatus* conidia

**Fig 2:**
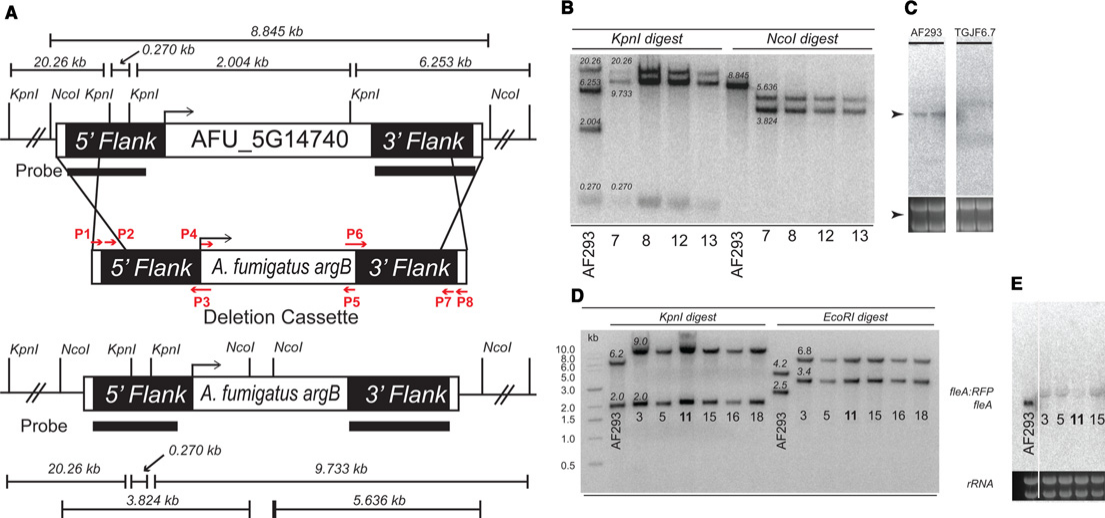
Generation of *ΔfleA* conidia in *A*. *fumigatus*. (A) Diagram showing the gene disruption cassette used to generate *ΔfleA* conidia. (B) Southern blot depicting successful deletion of *fleA*. The restriction digest pattern corresponding to deletion of *fleA* was used to identify those strains where *fleA* was deleted. (C) Confirmation of *fleA* deletion was provided by northern blotting; *fleA* transcript is visible in the WT strain AF293 whereas *fleA* deletion was confirmed by the absence of this FleA transcript in TGJF6.7, 6.8, and 6.12. (D) Confirmation of successful RFP tagging of *fleA* as shown by Southern blotting and northern blotting (E).

To further characterize *fleA* in *A*. *fumigatus* and examine the functional role of FleA binding to mucin, we created reagents to allow us to make quantitative measurements of *A*. *fumigatus* binding to mucin and to dissect the specific binding role of FleA. To facilitate visualization of the conidia, we created strains of *A*. *fumigatus* expressing nuclear GFP by transformation of AF293.1 and AF293.6 strains with the plasmid pJMP51 resulting in histone 2A fused GFP prototrophic (TJMP131.5) and auxotrophic (TGJF5.3) strains, respectively. TGJF5.3 was further modified and transformed to prototrophy using a disruption cassette targeting disruption of the *fleA* locus (Fig 2A) and multiple *ΔfleA* deletion transformants were identified. Confirmation of *fleA* deletion was demonstrated by Southern and northern blotting (Fig 2B and 2C). Despite repeated attempts to complement the *ΔfleA* deletion strains, we encountered problems such as phenotypic abnormalities in spore size and shape, which precluded generation of reliable complement reagents. Hence the mucin binding studies described below were repeated with several *ΔfleA* deletions to confirm the role of FleA as an *A*. *fumigatus* fucose binding protein. The mucin binding studies were also repeated using a novel synthetic glycomimetic inhibitor of FleA as an alternative experimental approach. To assess localization of FleA in conidia, we made a C-terminal RFP tagged *fleA* mutant in *A*. *fumigatus* by modifying the native *fleA* locus using a disruption cassette similar to that used to delete the *fleA ORF* (Fig 2D and 2E).

### Generation of *ΔfleA A*. *flavus* conidia

Multiple *ΔfleA* deletion *A*. *flavus* strains were generated using the same methods described for *A*. *fumigatus* above and as illustrated in S2 Fig.

### FleA is present on resting and swollen *A*. *fumigatus* conidia

**Fig 3:**
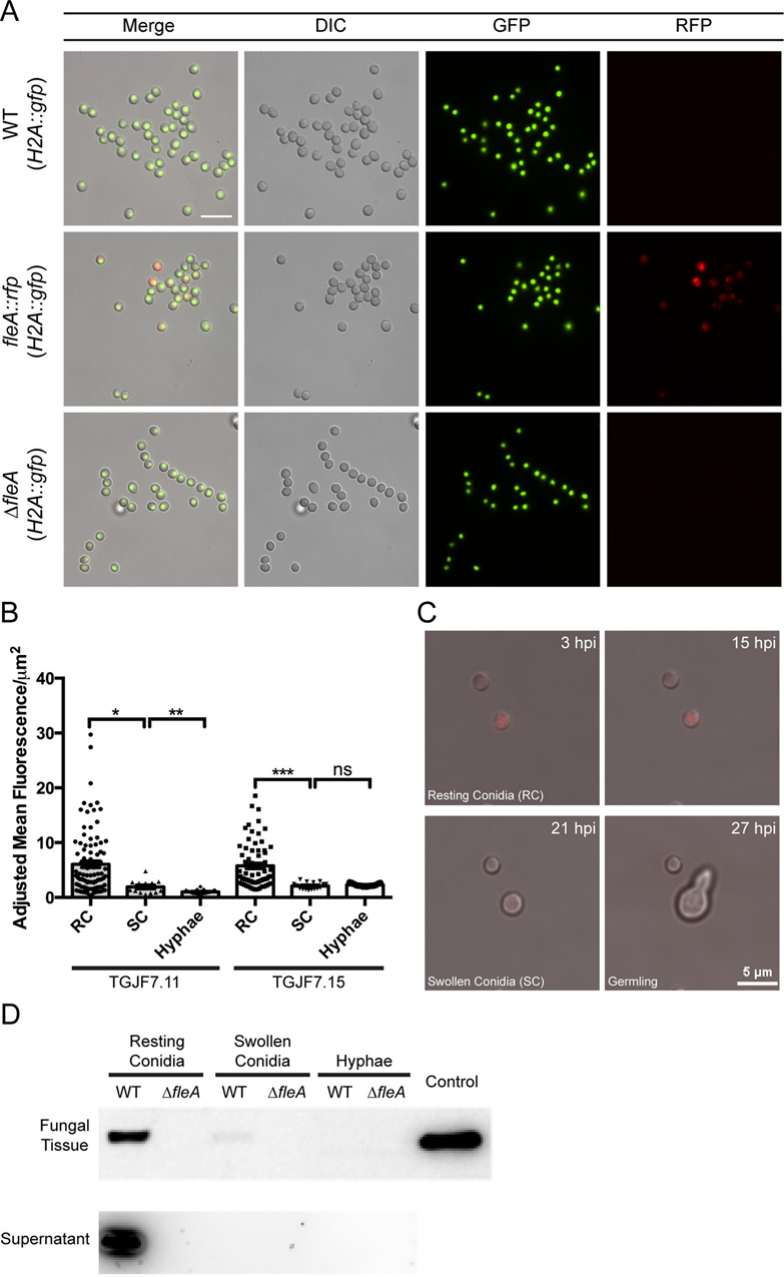
FleA is expressed in *A*. *fumigatus* conidia. (A) RFP-tagged FleA is present in *A*. *fumigatus* conidia when *fleA-rfp* is expressed under its endogenous promoter. No FleA signal is visible in the *ΔfleA* deletion conidia or the non-RFP-tagged WT conidia. Data is representative of at least 3 experiments. Scale bar is 10μM. (B) RFP-tagged FleA was quantified by fluorescence microscopy in 2 different strains (TGJF7.11, TGJF7.15) showing higher levels of FleA in resting conidia compared to swollen conidia and hyphae. (C) Still images showing RFP fluorescence during germination, at time points representative of resting and swollen conidia and hyphae. (D) Western blot image showing strong expression of FleA in resting WT conidia with weak expression in swollen conidia. Control lane shows recombinant FleA running at the expected molecular weight of 34kD. No protein was detected in hyphal extract or extracts from *ΔfleA* conidia (TGJF6.7). Secreted FleA was detected in 10x concentrated culture supernatants from resting conidia of WT only.

Protein levels of FleA on *A*. *fumigatus* conidia and hyphae have not been characterized. We found that RFP-tagged FleA is present on *A*. *fumigatus* conidia when *fleA-rfp* is expressed under its endogenous promoter (Fig 3A), and we used two different strains of FleA-RFP tagged conidia to quantify FleA levels over time by fluorescence microscopy (Fig 3B). FleA fluorescence was high on resting conidia (with some variation among conidia), low in swollen conidia, and largely absent in hyphae (Fig 3B and 3C). Time-lapse microscope images of germinating conidia show that FleA begins to decrease by 21 hours and is almost entirely absent by 27 hours (Fig 3C and S1 Movie). These results were confirmed in extracts from resting and swollen conidia and from hyphae that were analyzed for FleA protein by western blot (Fig 3D). We also investigated the possibility that FleA was secreted by conidia, even though FleA lacks a canonical secretion signal peptide according to the Signal P 4.0 server [27]. Analysis of proteins in concentrated culture supernatant of *A*. *fumigatus* at various developmental timepoints revealed that resting conidia shed significant amounts of FleA, whereas conditioned media from swollen conidia and hyphal culture supernatant did not have detectable FleA (Fig 3D).

### Binding of *A*. *fumigatus* conidia to airway mucins and phagocytosis by macrophages is FleA dependent

**Fig 4:**
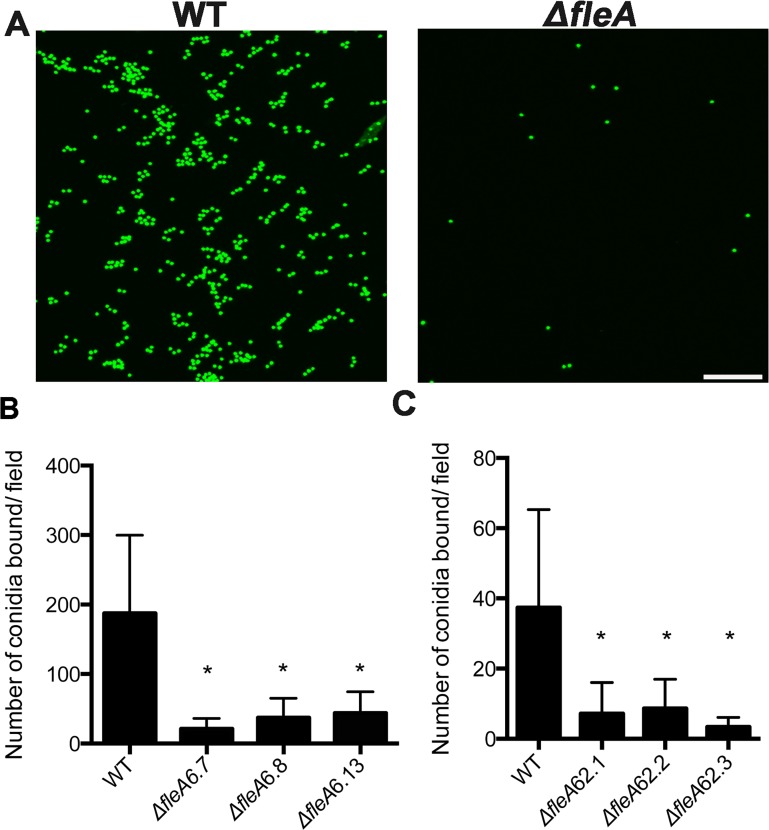
Binding of *Aspergillus* conidia to airway mucins is FleA dependent. (A) Z-stack confocal images showing binding of WT *A*. *fumigatus* conidia (TJMP 131.5) to mucin and that *ΔfleA* conidia have very limited binding. (B) Quantitative binding of WT *A*. *fumigatus* conidia to mucin or *ΔfleA* deletion mutants. (C) Quantitative binding of WT *A*. *flavus* conidia to mucin or *ΔfleA* deletion mutants. Note that *A*. *fumigatus*-mucin interactions were investigated using GFP labeled conidia whereas the *A*. *flavus*-mucin interactions were investigated using Calcofluor white-stained conidia (since the *A*. *flavus* conidia lack GFP). The data shown in panels B and C (27 replicates) reflects the mean ± SD of three independent experiments.

To examine if binding of *A*. *fumigatus* conidia to airway mucins is FleA dependent, we first developed a binding assay to determine the specific binding role of FleA. For the conidia-binding assay, we coated mucins onto chamber slides and used confocal microscopy to image binding of conidia. As shown in Fig 4A and 4B, the binding of three independent *ΔfleA* deletion mutant strains (TGJF 6.7, 6.8 and 6.13) to mucin is much weaker than the binding of WT conidia. These data demonstrate that *A*. *fumigatus* conidia bind to mucin in a FleA-dependent manner. After confirming that all 3 *ΔfleA* mutant strains showed identical patterns in mucin binding assays, we chose the *A*. *fumigatus* strain TGJF6.7 for all further functional studies.

### Binding of *A*. *flavus* conidia to airway mucins is FleA dependent

To determine if FleA is also required for binding of *A*. *flavus* conidia to mucin, we examined the binding of WT and *ΔfleA A*. *flavus* conidia in our chamber slide assay. As shown in Fig 4C, the binding of three independent *ΔfleA* deletion mutant strains of *A*. *flavus* (TFYL 62.1, 62.2 and 62.3) to mucin is much weaker than the binding of WT conidia.

**Fig 5:**
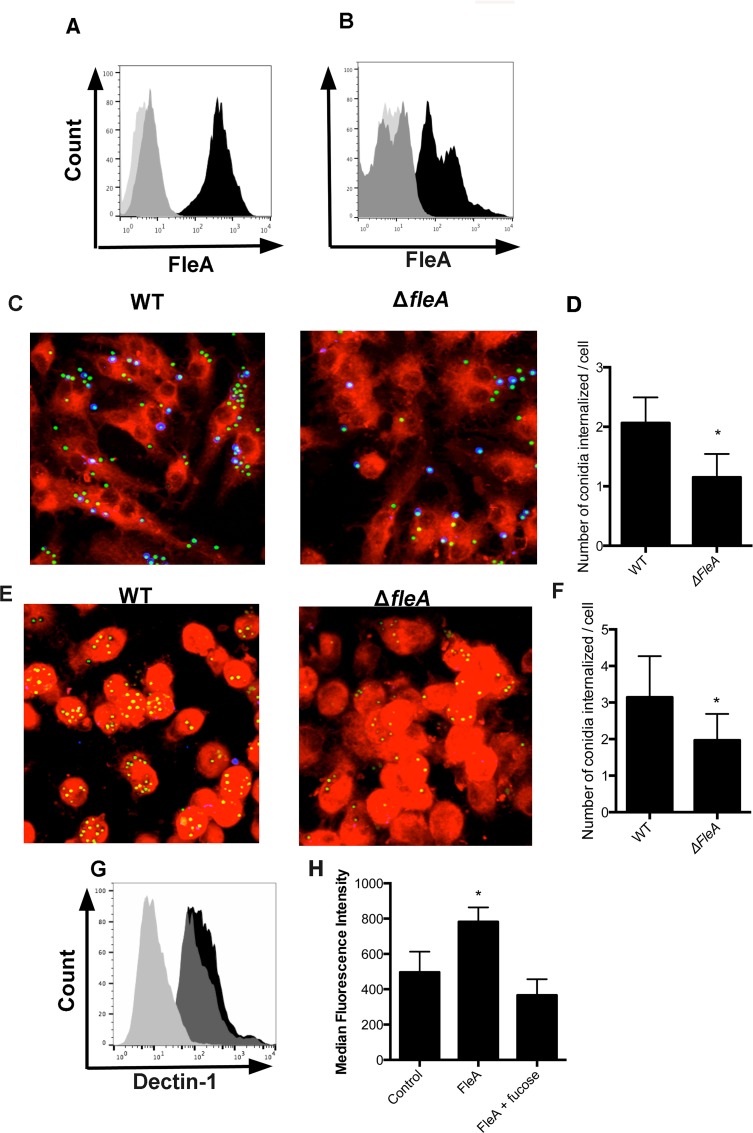
Binding of *A*. *fumig*atus conidia to alveolar macrophages is FleA-dependent. FACS data showing binding of FleA (black) to (A) RAW264.7 or (B) primary human alveolar macrophages. 100mM fucose (dark grey) inhibits binding almost down to the background level (light grey). Z-stack confocal images show binding and phagocytosis of GFP expressing WT conidia by (C) RAW264.7 cells, (E) primary human alveolar macrophages and that *ΔfleA* conidia are not bound well or phagocytosed effectively. Cells were dyed with CellMask Deep red (red); internalized conidia express GFP (green); calcofluor white (non-internalized conidia) stain blue. Quantitative data (expressed as an index of total number of conidia internalized/total cell number) demonstrate how loss of FleA significantly inhibits binding and phagocytosis of *A*. *fumigatus* conidia by (D) RAW264.7 cells or (F) primary human alveolar macrophages. Calcofluor white stained conidia were excluded from these counts to reflect only internalized conidia. The data shown in D (6 replicates) reflects the mean ± SD of three independent experiments whereas the data in panel F (6 replicates) reflects the mean ± SD of three independent donors. (G) FACS plots showing that binding of Dectin-1 is not significantly different between WT (dark grey) and *ΔfleA* (black) conidia compared to control (light grey). (H) Binding and internalization of FleA coated microspheres by RAW264.7 cells is significantly higher than control and inhibited by 500mM fucose. *Denotes significantly different from control, p = <0.05. Scale bar is 10μM.

Fucosylated glycoproteins are not restricted to mucin glycoproteins. Macrophages also express multiple fucosylated proteins on their cell surface, including membrane-tethered mucins [24]. We therefore explored whether *fleA* expression by *A*. *fumigatus* might mediate binding or phagocytosis by macrophages. We first explored binding of recombinant FleA to macrophages by flow cytometry and showed that FleA binds very strongly to the surface of RAW264.7 mouse macrophages and primary human alveolar macrophages in a fucose-dependent manner (Fig 5A and 5B). Next, we investigated phagocytosis of *A*. *fumigatus* conidia to RAW264.7 mouse macrophages. Using confocal microscopy, we found that *A*. *fumigatus* WT conidia bound to RAW264.7 cells and that many are internalized/phagocytosed (Fig 5C). Notably, the internalization of *ΔfleA* conidia was 50% less than that of WT (Fig 5C and 5D). We repeated these experiments in alveolar macrophages isolated from bronchoalveolar lavage from healthy human subjects. Similar to data from the RAW264.7 cells, we found that *A*. *fumigatus* conidia are internalized and phagocytosed by human macrophages and that *ΔfleA* conidia show a reduction in phagocytosis of 40% compared to WT (Fig 5E and 5F). Thus, fucosylated structures on lung macrophages act as receptors for *A*. *fumigatus* FleA, resulting in enhanced phagocytosis of conidia.

To rule out the possibility that deletion of FleA causes changes to β-glucan in the fungal cell wall that could be affecting binding and phagocytosis, we used FACS to examine the binding of a β-glucan ligand (Dectin-1) to *ΔfleA* and WT conidia. We found no significant differences in the affinity of recombinant biotinylated Dectin-1 for *ΔfleA* and WT conidia in these experiments (Fig 5G *)*. We also coated fluorescent microspheres with FleA to create a simplified model of conidia and found that FleA causes a significant and fucose-dependent increase in binding and phagocytosis of microspheres by RAW264.7 cells (Fig 5H).

### (2E)-hexenyl α-L-fucopyranoside is a potent functional inhibitor of FleA

**Fig 6:**
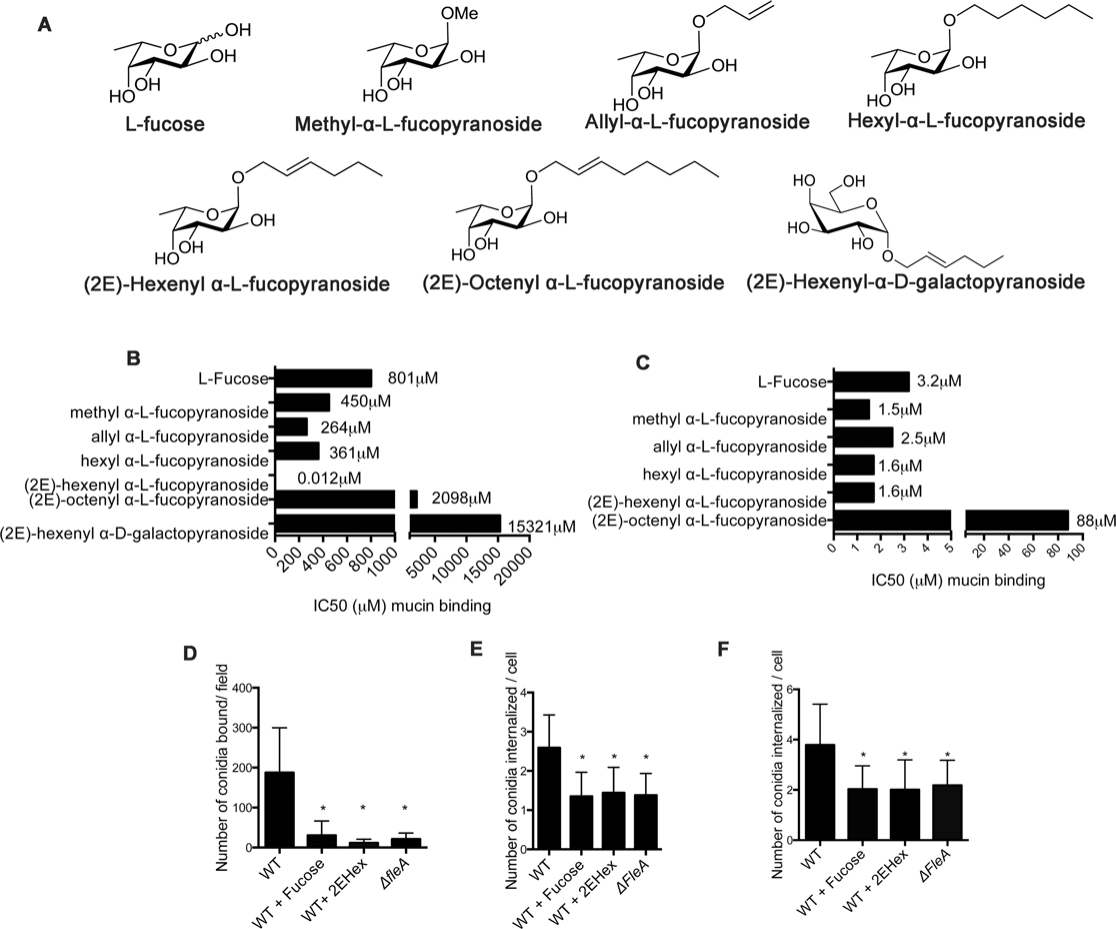
Inhibition of FleA by 2EHex or fucose results in a loss of mucin binding and greatly reduced phagocytosis by macrophages. (A) Structures of fucopyranoside compounds. (B) Amount of compound required to inhibit binding of labeled FleA to mucin by 50% (IC50 [μM]). Addition of a methyl or allyl group to the anomeric position of fucose (methyl α-L-fucopyranoside, allyl α-L-fucopyranoside) improves inhibition by 2–4 fold but inclusion of a longer (6-carbon) unsaturated chain improves inhibition by 3 orders of magnitude. Removing the double bond or extending the carbon chain beyond 6 carbons (hexyl α-L-fucopyranoside, (2E)-octenyl α-L-fucopyranoside) markedly decreases inhibition. (2E)-hexenyl α-D-galactopyranoside has no effect on FleA binding to mucin. (C) Amount of compound required to inhibit PAIIL binding to mucin by 50% (IC50 [μM]). 2EHex does not have a strong inhibitory effect on PAIIL-mucin interactions. (D) Inhibiting WT conidia with 10mM 2EHex or 100mM fucose significantly reduced binding of conidia to mucin. WT conidia were poorly phagocytosed in the presence of 2EHex or fucose by RAW264.7 cells (E) or primary human macrophages (F). The data shown in D and E (6 replicates) reflects the mean ± SD of three independent experiments whereas the data in panel F (6 replicates) reflects the mean ± SD of three independent donors. *Denotes significantly different from control, p = <0.05.

To provide additional evidence that binding of *A*. *fumig*atus conidia to mucins and alveolar macrophages is FleA-dependent, we synthesized a library of modified fucopyranoside structures using methods we previously described for inhibitors of the FimH lectin in *E*. *coli* [28] (Fig 6). To screen for the relative potency of different fucopyranosides, we used the FleA-mucin binding assay. In this way, we found that (2E)-hexenyl α-L-fucopyranoside (2EHex) (Fig 6A) inhibits FleA with marked (nanomolar) potency (Fig 6B). In contrast to its potent inhibition of FleA binding to mucin, 2EHex does not show potent inhibition of PA-IIL (a fucose-binding lectin from *Pseudomonas aeruginosa*) binding to mucin (Fig 6C). We next tested the effects of 2EHex and fucose on interactions between *A*. *fumigatus* conidia and mucins and macrophages. We found that both 2EHex and fucose decreased the mucin binding of WT *A*. *fumigatus* conidia to the levels observed with *ΔfleA* conidia (Fig 6D). 2EHex also decreased the phagocytosis of WT *A*. *fumig*atus conidia by RAW264.7 cells and primary human macrophages to the levels observed with the *ΔfleA* conidia (Fig 6E and 6F). These inhibition studies confirm that binding of *A*. *fumigatus* conidia to mucins and macrophages requires the fucose binding activity of FleA.

### FleA loss increases lung infection and lung injury by *A*. *fumigatus*

**Fig 7:**
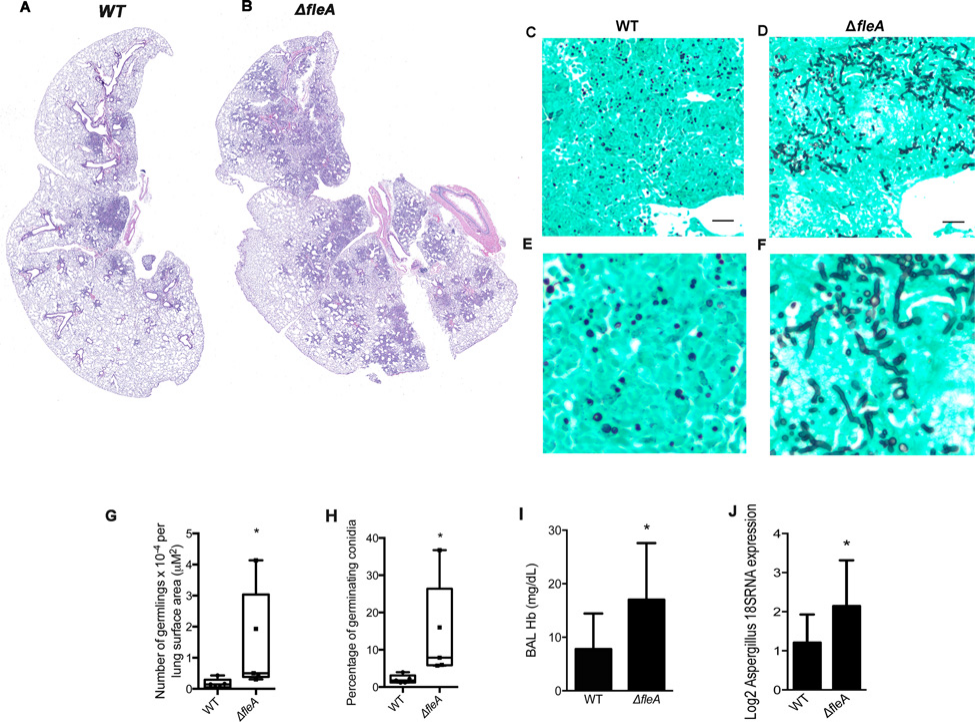
Mice infected with FleA-deficient *A*. *fumigatus* conidia have more severe *Aspergillus* lung infection. (A). Section of whole mouse lung after infection with WT conidia. (B). Section of whole mouse lung after infection with *ΔfleA* conidia. (C)(D) GMS stained sections (20x image) of mouse lung after intranasal infection with WT conidia or *ΔfleA* conidia. (E)(F) GMS stained sections (zoom image) of mouse lung after intranasal infection with WT conidia or *ΔfleA* conidia. (G). Germination of A. *fumigatus* conidia in the lungs of mice infected with WT or *ΔfleA* conidia. The tissue is stained with GMS and the number of germinating conidia are quantified in at least 20 high power fields per mouse using lung surface area (μM^2^) as a reference (n = 5 mice per group). (H) The percentage of germinating *A*. *fumigatus* conidia in the lungs of mice infected with WT or *ΔfleA* conidia. Germinating conidia are significantly more prevalent in the lungs of mice infected with *ΔfleA* conidia (n = 5 mice per group). (I) Hemoglobin concentration is significantly higher in lung lavage from mice infected with *ΔfleA* conidia than WT conidia (n = 10 mice per group) (J). Gene expression for *Aspergillus* 18S is significantly higher in lung homogenates from mice infected with *ΔfleA* conidia than from mice infected with WT conidia (n = 10 per group). All mouse samples were harvested 3 days after infection. Scale bar is 10μM. Data are mean +/- SD. *Denotes significantly different from control, p = <0.05.

Based on our data that FleA is required for binding and phagocytosis of conidia by macrophages, we hypothesized that *ΔfleA* conidia might evade phagocyte killing leading to increased lung infection compared to WT. To test this hypothesis, we infected immunocompetent C57BL/6 mice intranasally with WT conidia or *ΔfleA* conidia. H&E staining of infected lung tissue showed that mice infected with WT conidia had well-contained pneumonia (Fig 7A) whereas *ΔfleA* conidia treated animals had a poorly contained pneumonia (Fig 7B). GMS staining showed limited numbers of conidia with little evidence of germination in sections from WT treated animals (Fig 7C and 7E). In contrast, a large number of conidia from *ΔfleA* treated animals showed hyphae generation (germlings) evident on the GMS stain, typical of invasive aspergillosis (Fig 7D and 7F). Both the total number and percentage of germinating conidia are significantly higher in mice infected with *ΔfleA* conidia than WT conidia (Fig 7G and 7H). Lung injury is more severe in *ΔfleA*-infected mice, as evidenced by a higher concentration of hemoglobin in bronchoalveolar lavage (BAL)(Fig 7I). *Aspergillus* 18S gene expression in lung homogenates from *ΔfleA*-infected mice is higher than in lung homogenates from WT infected mice, indicative of higher fungal burden (Fig 7J).

**Fig 8:**
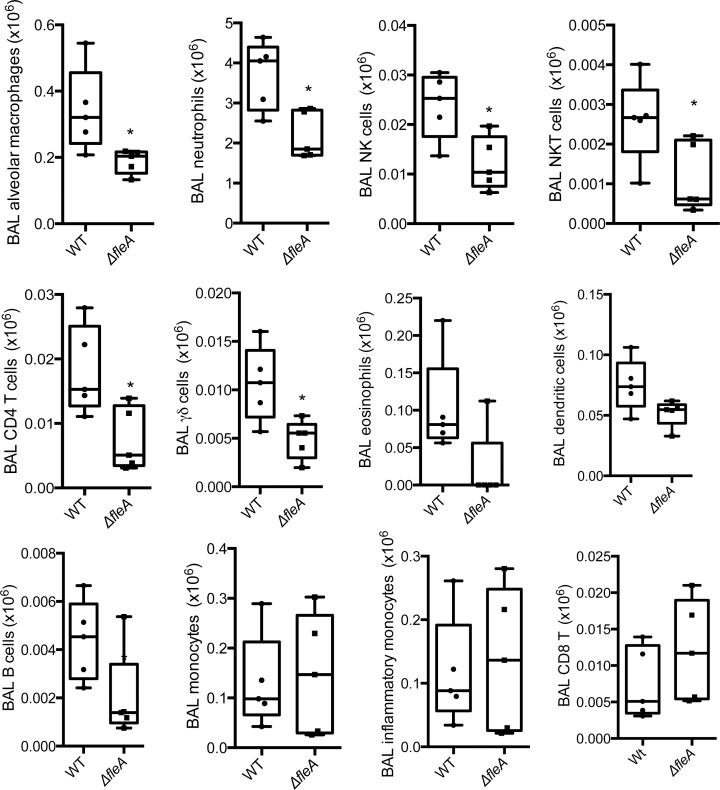
*ΔfleA* conidia treated animals have more severe lung injury and reduced recruitment of some immune cell types. FACS-based identification and quantification of immune cells in bronchoalveolar lavage of mice infected with WT conidia or *ΔfleA* conidia (n = 5 per group). All mouse samples were harvested 3 days after infection.

BAL cells from mice infected with WT and *ΔfleA* conidia were analyzed by flow cytometry to quantify multiple immune cells and by multiplex immunoassay to quantify multiple cytokines, chemokines and growth factors. Mouse BAL cells were labeled with a panel of antibodies including CD11c, F4/80, CD11b, MHCII, Ly6G, Ly6C, NK1.1, TCRβ, B220, CD4 and CD8. Compared to mice infected with WT conidia, we found that several cell types were decreased in BAL from mice infected with *ΔfleA* conidia, including alveolar macrophages, neutrophils, NK and NKT cells and CD4 and γΔ T cells (Fig 8). No significant differences were observed for eosinophils, dendritic cells, B cells, monocytes and inflammatory monocytes and CD8 T cells. Compared to mice infected with WT conidia, we found that the concentrations of multiple cytokines, chemokines and growth factors were similar in BAL from mice infected with *ΔfleA* conidia, including IL-6, IFNγ, KC and VEGF in BAL fluid (S1 Table).

## Discussion

We have discovered that FleA produced by *A*. *fumigatus* conidia allows airway mucins to bind conidia and macrophages to effectively phagocytose them. Notably, when we engineer conidia that lack FleA, the resultant *ΔfleA* conidia show increased virulence in a mouse model of *A*. *fumigatus* pneumonia. Together these data uncover a novel mechanism of host defense against *A*. *fumigatus* infection in which fucosylated receptors in the airway mucus gel and on the surface of macrophages bind FleA to hasten the elimination of *A*. *fumigatus* conidia.

To date, research on binding of *A*. *fumigatus* conidia to lung proteins has focused on binding to basement membrane proteins in the airway epithelium [29–32]. But mucins provide the first line of defense against inhaled pathogens in the bronchi and bronchioles [25], and studies of binding of *A*. *fumigatus* conidia to human airway mucins are highly relevant to mechanisms of infection and invasion. Mucin glycans include multiple fucosylated structures [22], and we report avid binding of FleA to human airway mucins that is inhibited by a wide range of fucose structures, including fucose in α1,2, α1,3, α1,4, or α1,6 linkages. We also report that deletion of FleA in *Aspergillus fumigatus* conidia markedly decreases mucin binding and that this role for FleA is conserved in the pathogenic *A*. *flavus*. We conclude that a range of sterically available fucosylated glycans in mucins can act as FleA ligands and the airway mucus gel is a powerful “sticky” barrier that can capture and remove conidia to guard against invasive infection.

Phagocytosis of inhaled conidia by alveolar macrophages represents an important innate immune defense against *A*. *fumigatus* infection, especially in the alveolar lung compartment where mucocilliary clearance does not operate. We explored the role of FleA in phagocytosis of *A*. *fumigatus* conidia by murine and human macrophages using two approaches. First, we compared the phagocytosis of wild type (WT) conidia and *fleA-*deficient *(∆fleA*) conidia and showed that macrophage phagocytosis of the *∆fleA* conidia is decreased by 40–50%. Second, we screened a library of fucopyranosides to reveal that (2E)-hexenyl α-L-fucopyranoside (2EHex) inhibits FleA with nanomolar efficacy and also inhibits the binding and phagocytosis of WT *A*. *fumigatus* conidia by lung macrophages. Together, these two lines of evidence leads us to conclude that *A*. *fumigatus* conidia interact with macrophages in a mechanism that requires the fucose binding activity of FleA. Multiple glycoproteins on the surface of macrophages could act as the receptors for FleA, including membrane-tethered mucins [24]. Neutrophils are also important in cellular immunity against *Aspergillus* [33, 34], but we did not study whether neutrophils have defective uptake of *fleA*-deficient conidia, and it remains unknown whether fucosylated receptors on neutrophils have a role in host defense against *Aspergillus*. The identity of all of the cell types participating in FleA-dependent host defense in the lung has not been elucidated in this study. While we have described an alveolar macrophage dependent mechanism, it is also likely that other non-alveolar macrophage dependent cellular mechanisms, including neutrophils or monocytes, can contribute to FleA mediated host defense.

The FleA-dependent binding of *A*. *fumigatus* conidia to mucin and the poor uptake of *ΔfleA* conidia by lung macrophages indicate that FleA is not a virulence factor. Instead, we propose it is a fungal protein recognized by the host to promote defense against invasive aspergillosis. We provide evidence for this in our experiments in which we infected immunocompetent mice with *A*. *fumigatus* WT or *ΔfleA* conidia. The mice infected with the *ΔfleA* conidia developed invasive aspergillosis whereas those infected with WT conidia did not. Notably, the *ΔfleA* infected mice showed blunted recruitment of immune cells integral to the fungal immune response. Specifically, FACS analysis of BAL cells showed a significant decrease in the number of alveolar macrophages, neutrophils, NK and NKT cells and CD4 and γΔ T cells. Thus, it appears that *ΔfleA* conidia fail to elicit the same inflammatory response as WT conidia resulting in reduced recruitment of key effector cells in the fungal immune response in the lung. We conclude that the absence of FleA in *A*. *fumigatus* conidia results in a hypo-inflammatory response and promotes invasive infection.

Our experiments prove that FleA is a pathogen-associated molecular pattern that can be recognized by lung mucins and macrophages to protect the host from infection. The fact that we show that *A*. *flavus* also uses FleA to bind lung mucin and that phylogenetic analysis reveals FleA present in several pathogenic species may suggest a conserved role for FleA in fucose-mediated host pathogen interactions. It is unclear why *A*. *fumigatus* and other pathogenic fungi have evolved to express *fleA*. Since fungi commonly grow on carbon-rich carbohydrate substrates, it is possible that FleA is involved in helping these organisms to establish a niche on the surface of carbohydrate-rich substrates.

Our finding for a role for FleA-fucose mediated mechanism of host defense against *A*. *fumigatus* does not cast any doubt on the validity of the well established role for β-glucan/Dectin-1 interactions [35]. β-glucan is abundant on swollen conidia and hyphae, and Dectin-1 binds β-glucan on swollen conidia to trigger a robust host defense response [36]. But Dectin-1 does not bind to resting conidia, because these conidia have masked exposure of β-glucans due to the action of hydrophobin proteins [37, 38] and the mechanism of clearance of resting conidia from the lung is not completely understood. We propose a model whereby fucosylated receptors on mucins and macrophages interact with FleA on resting conidia to facilitate clearance of resting conidia via the mucociliary escalator and/or macrophage ingestion. Conidia that escape this initial response and mature (swell and germinate), possibly through the shedding of FleA, would be cleared by Dectin-1 mediated phagocytosis [9]. Our data thus reveal contrasting roles for distinct protein carbohydrate interactions in host immune responses to *A*. *fumigatus*. On one hand, a macrophage expressed lectin—Dectin-1—recognizes and binds a carbohydrate structure (β-glucan) on *A*. *fumigatus* conidia to promote phagocytosis and killing. On the other hand, we reveal here that fucosylated carbohydrates on macrophages engage a *conidial* lectin (FleA) to promote phagocytosis and killing. We conclude that protein- and carbohydrate-based defenses on macrophages provide complimentary mechanisms to prevent potentially fatal *Aspergillus* lung infections.

## Methods

### Subjects and clinical samples

Induced sputum was collected from 5 healthy nonsmoking, non-allergic subjects aged between 24–55 years (4 male) as described [39]. Bronchoalveolar lavage (BAL) was collected from 3 healthy non-smoking, non-allergic subjects aged 30–45 years (2 male, 1 female) by instilling 4 aliquots of 50mLs of warmed (37°C) normal saline into a segmental bronchus in the right middle lobe or lingula. After the first two 50mL aliquots were instilled and aspirated from one segmental bronchus, the bronchoscope was moved to an adjacent bronchus in the same segment for collection of two additional 50 mL aliquots.

### Purification of high molecular weight mucin from sputum

8M guanidine hydrochloride was added in 1:1 volume to sputum samples and the samples rotated at 4°C until homogenized. Mucins were then purified from the sputum as described previously [40, 41] with additional details in S1 Text.

### FleA binding to mucin

Recombinant FleA (prepared as described previously [18]) was biotinylated with EZ-link sulfo NHS biotin (Pierce, Thermo Fisher, Rockford, IL). Purified human mucin was coated on a Nunc maxisorp plate at 20 μg/ml in carbonate bicarbonate buffer pH 9.6 overnight at 4°C, washed and blocked with TBS + 0.05% Tween-20, 10mM CaCl_2_, 3% BSA. Biotinylated recombinant FleA was incubated at 5 μg/ml in TBS + 0.05% Tween-20, 10 mM CaCl_2_, 1% BSA (binding buffer) in the presence or absence of 100mM L-fucose or 100mM L-galactose. For inhibition assays, recombinant FleA was incubated with a dilution series of synthesized carbohydrate compounds starting at 5mM. Plates were washed with binding buffer, incubated with ExtrAvidin-alkaline phosphatase (Sigma-Aldrich, St Louis, MO) and detected using phosphatase substrate (Sigma-Aldrich, St Louis, MO) in carbonate bicarbonate buffer pH 9.6 + 1 mM MgCl_2_ and read at 405nm (Biotek Synergy plate reader, Winooski, VT).

### Synthesis of carbohydrates

The disaccharides were synthesized by glycosylating appropriately protected glucose acceptors with a fucosyl bromide donor, 2,3,4-tri-O-benzyl-L-fucopyranosyl bromide, using Lemieux’s halide assisted conditions [42], followed by deprotection of the α-linked disaccharides using catalytic hydrogenolysis to give the target structures. Detailed synthesis methods are in S1 Text.

### Aspergillus strains and culture (S2 Table)

Unless noted, all *A*. *fumigatus* strains were propagated on solid glucose minimal media (GMM) at 37°C [43]. *A*. *fumigatus* asexual spore suspensions were fixed, where appropriate, in 4% formaldehyde in PBS.

### Generation of FleA mutant conidia

*A*. *fumigatus* strains expressing GFP were constructed using pJMP51 to transform AF293.1 and AF293.6 which yielded TJMP131.5 (*GFP*::*H2A*) and TGJF5.3 (*GFP*::*H2A*, *argB1*), respectively. A *fleA* gene disruption cassette (Fig 2A) was used to transform TGJF5.3 to create TGJF6.7, 6.8 and 6.13 (*GFP*::*H2A*, *∆fleA*). The strain was confirmed by Southern and northern analysis (Fig 2B and 2C). TGJF5.3 was also transformed with a FleA RFP (*fleA*:*RFP*) tagged cassette yielding the prototrophic *fleA*:*RFP* strain, TGJF7.11. FleA tagging was confirmed microscopically and by Southern and northern analysis Fig 2D and 2E). *A*. *flavus ΔfleA* deletion mutants were created by transforming the deletion construct into parental strain CA14*∆ku70∆pyrG* [44] to create strains TFYL62.1–62.3. Single integration of the deletion cassette was verified via Southern analysis (S2 Fig). More detailed methods are available in S1 Text.

### Immunofluorescence of *Aspergillus* conidia

*A*. *fumigatus* strains were cultured on GMM at 37°C for 3 days, spores were harvested, placed on a pre-cleaned glass slide and coverslipped. Images were taken of GFP and RFP fluorescence using a Nikon Ti inverted microscope equipped with a Nikon Plan Apo VC 60x/1.40 Oil DIC/∞/0.17 WD. Time-course microscopy was carried out over 27 hours at 37°C. The average fluorescent intensity at each developmental state (resting conidia, swollen conidia, and hyphae) of untagged FleA (TJMP131.5 or wild type) was subtracted from the mean fluorescent intensity value of two different transformants (TGJF7.11 and TGJF7.15) expressing RFP-tagged versions of FleA. The adjusted mean fluorescence was then standardized to area.

### Western blot of *A*. *fumigatus* extracts and supernatant

Resting and swollen *A*. *fumigatus* conidia and hyphae were isolated from WT and *ΔfleA* cultures as described in S1 Text and extracted in 50mM Tris/HCl pH 7.4, 50 mM EDTA, 2% SDS, and 40 mM β-Mercaptoethanol. Protein concentrations were determined by BCA assay and 15 μg of protein was loaded into each well of a 4–12% BOLT SDS PAGE gel (Life Technologies, Grand Island, NY) and electrophoresed. Gel was then blotted onto nitrocellulose, blocked with non fat milk and stained with an anti-FleA rabbit polyclonal antibody [18] and donkey anti-rabbit HRP (Jackson immunoresearch, West Grove, PA) prior to chemiluminescent detection. Culture supernatants were filtered through a 0.2 μM filter and concentrated 10x in a 0.5 ml Amicon Ultra (EMD Millipore, Billirica, MA) before being run as described above.

### Mucin conidia binding assay

8 well glass chamber slides (Labtek, Scotts Valley,CA) were coated with 20 μg/ml purified human mucin in dH_2_O overnight at 37°C and then blocked in PBS + 1% BSA for 1 hour. Fixed *A*. *fumigatus* conidia suspensions were centrifuged at 6000 x g for 5 minutes to pellet and resuspended in PBS + 1% BSA in the presence or absence of 10mM (2E)-hexenyl α-L-fucopyranoside (2EHex) or 100mM fucose. 2x10^7^ conidia were added per well and incubated for 4 hours at room temperature. Unbound conidia were removed by washing in PBS+ BSA, the slides were mounted in Prolong Gold anti-fade reagent (Life Technologies, Grand Island, NY) and allowed to cure for 24 hours prior to sealing. Images were acquired using an FV10i confocal microscope (Olympus, Center Valley, PA) using the multipoint Z-stack mode to acquire 9 fields per well with 3 wells imaged per condition per experiment. Each Z-stack image was compressed into a single plane of focus and conidia were counted using NIH Image J with the ITCN plugin. Each experiment was repeated at least 3 times. *A*. *flavus*-mucin interactions were investigated as described above with one exception. These conidia lack GFP so were stained with Calcofluor white for 5 minutes to allow imaging prior to adding to mucin-coated slides.

### Cell culture

RAW 264.7 cells (UCSF cell culture facility) were maintained in DMEM + 10% fetal bovine serum + 1% penicillin/streptomycin until seeded and grown on 8 well chamber slides (Labtek, Scotts Valley, CA) overnight. Human alveolar macrophages from BAL were centrifuged at 450 x g for 10 minutes and washed with PBS prior to plating on poly-L-lysine coated 8 well chamber slides in RPMI 1640+ 10% fetal bovine serum + 1% penicillin/streptomycin + 0.5μg/ ml amphotericin B. Cells were washed after 2 hours of adherence and cultured overnight prior to experiments.

### FleA binding to macrophages by flow cytometry

RAW264.7 or primary human lung macrophages were incubated with Alexa-488 tagged recombinant FleA in the presence or absence of 100mM fucose prior to analysis on a Becton Dickenson FACScalibur and Flow Jo software (Treestar, Ashland, OR).

### Phagocytosis assay

RAW 264.7 cells were plated at 5x10^4^/well on 8 well chamber slides (Labtek, Scotts Valley, CA) and allowed to grow overnight in culture media. 5x10^6^ conidia from either the PFA-fixed WT or *ΔfleA* strain were added per well in the presence or absence of 10mM 2EHex or 500mM fucose and incubated at 37°C for 1 hour. Wells were washed and incubated for a further 2 hours at 37°C for complete uptake. Cells were stained with 7.5μg/ml CellMask Deep Red plasma membrane stain (Life Technologies, Grand Island, NY) and calcofluor white (Sigma, St Louis, MO), washed with PBS and mounted with Fluoromount-G (Southern Biotech, Birmingham, AL). Z-stack images were acquired using an FV10i confocal microscope (Olympus, Center Valley, PA). Each Z-stack image was compressed into a single plane of focus and both internalized conidia and cell number were counted using NIH Image J with the cell counter plugin. Phagocytic index was calculated as the number of conidia internalized per cell. Internalized conidia were counted as conidia within the boundary of the cell that were not stained with calcofluor white. Calcofluor white stained conidia were excluded from the count as they were not internalized. For human macrophages, cells were plated at 5x10^5^ per well and grown overnight. 1.5x10^6^ conidia from the PFA fixed WT or *ΔfleA* strains were added per well in the presence or absence of 10mM 2EHEX, incubated at 37°C for 30 minutes then washed, stained and mounted.

### Flow cytometry analysis of *A*. *fumigatus* conidia

Recombinant Dectin-1 was purchased from R&D Systems (Minneapolis, MN) and biotinylated using the EZ-link sulfo NHS biotin kit (Thermo Fisher Waltham, MA) according to manufacturers recommendations. 1x10^7^ WT or *ΔfleA* conidia were labeled with biotinylated Dectin-1 and Streptavidin-PE (Biolegend, San Diego, CA) and fixed in 4% paraformaldehyde prior to analysis on a Becton Dickenson FACSCalibur and FlowJo software (TreeStar, Ashland, OR).

### Phagocytosis of FleA-coated particles

Yellow-green 1μM sulfate treated FluoSpheres (Life Technologies, Grand Island, NY) were coated with recombinant FleA, added to RAW264.7 cells in the presence or absence of 500mM fucose and incubated with agitation for 2 hours at 37°C prior to extensive washing with DMEM to remove unattached FluoSpheres from the cell surface. Cells were fixed in 4% paraformaldehyde prior to analysis on a Becton Dickenson FACSCalibur and FlowJo software (TreeStar, Ashland, OR).

### Mice

Male C57BL/6 mice from Charles River Laboratories (Wilmington, Massachusetts, MA) were housed in a pathogen free facility at UCSF. Animal experiments followed protocols approved by the UCSF Institutional Animal Care and Use Committee.

### Mouse model of *Aspergillus* infection

Eight to ten week old C57BL/6 mice were infected intranasally with 5x10^7^ of WT or *ΔfleA* conidia. Mice were sacrificed 3 days post infection. Measures of lung inflammation, infection and lung injury were evaluated using methods described in S1 Text.

### Statistical methods

Data analyses were performed GraphPad Prism version 6 (GraphPad, San Diego, CA). ANOVA was used for three-group comparisons followed by pairwise analyses with the Tukey multiple comparisons test when appropriate. Two group comparisons were analyzed using the Students t-test or for non-parametric analyses, a ranked Mann-Whitney test.

### Ethics statement

Human samples were obtained from the UCSF Airway Tissue Bank (ATB). All participants signed two informed consent forms—one for the original study protocol and the other for the ATB protocol. All study and ATB procedures were reviewed and approved by the UCSF Committee on Human Research, protocol number 11–05176. All animal studies were carried out in strict accordance with the recommendations in the Guide for the Care and Use of Laboratory Animals of the National Institute of Health. All protocols involving animals were approved by the animal care and use committee at UCSF and are in compliance with Public Health Service Policy (PHS), (IACUC protocol number AN090458-03).

### Accession numbers

FleA, A. fumigatus, NC_007198.1, XP_753183.1

FleA, A. flavus, NW_002477244.1, XP_002380155.1

PAIIL, NC_002516.2 NP_252051.1

Dectin-1, NM_022570.4, NP_072092.2

MUC5AC, NM_001304359.1, NP_001291288.1

MUC5B, NM_002458.2, NP_002449.2

## Supporting Information

S1 Fig Phylogenetic analysis of fucose-binding lectins using FastTree Neighbor-Joining method. *Aspergillus* and bacterial fucose-binding lectins are highlighted in green and orange boxes, respectively. *Aleuria aurantia* lectin (AAL) was used to BLAST fungal and bacterial fucose-binding lectin protein sequences deposited in NCBI (blue box). A multiple sequence alignment of the selected sequences was used to identify the conserved region among bacterial and fungal sequences, which was extracted and subsequently used for phylogenetic analysis. Bootstrap values are presented at nodes.
(TIF)

S2 Fig Generation of *ΔfleA* conidia in *A*. *flavus*. Southern blot depicting successful deletion of *fleA* in *A*. *flavus*
(TIF)

S1 Movie Movie examining RFP-tagged FleA protein levels over time. (M4V)

S1 Table Luminex assay in BAL from WT and *ΔfleA* treated animals. (PDF)

S2 Table Strains of *A*.*fumigatus* used. (PDF)

S3 Table Primers used in this study. (PDF)

S1 Text Supplemental materials and methods. (DOCX)
