# Supplementary figures and images for: FleA Expression in Aspergillus fumigatus Is Recognized by Fucosylated Structures on Mucins and Macrophages to Prevent Lung Infection
Source: PLoS Pathog. 2016 Apr 8;12(4):e1005555. doi: 10.1371/journal.ppat.1005555 (PMC4825926; doi:10.1371/journal.ppat.1005555)

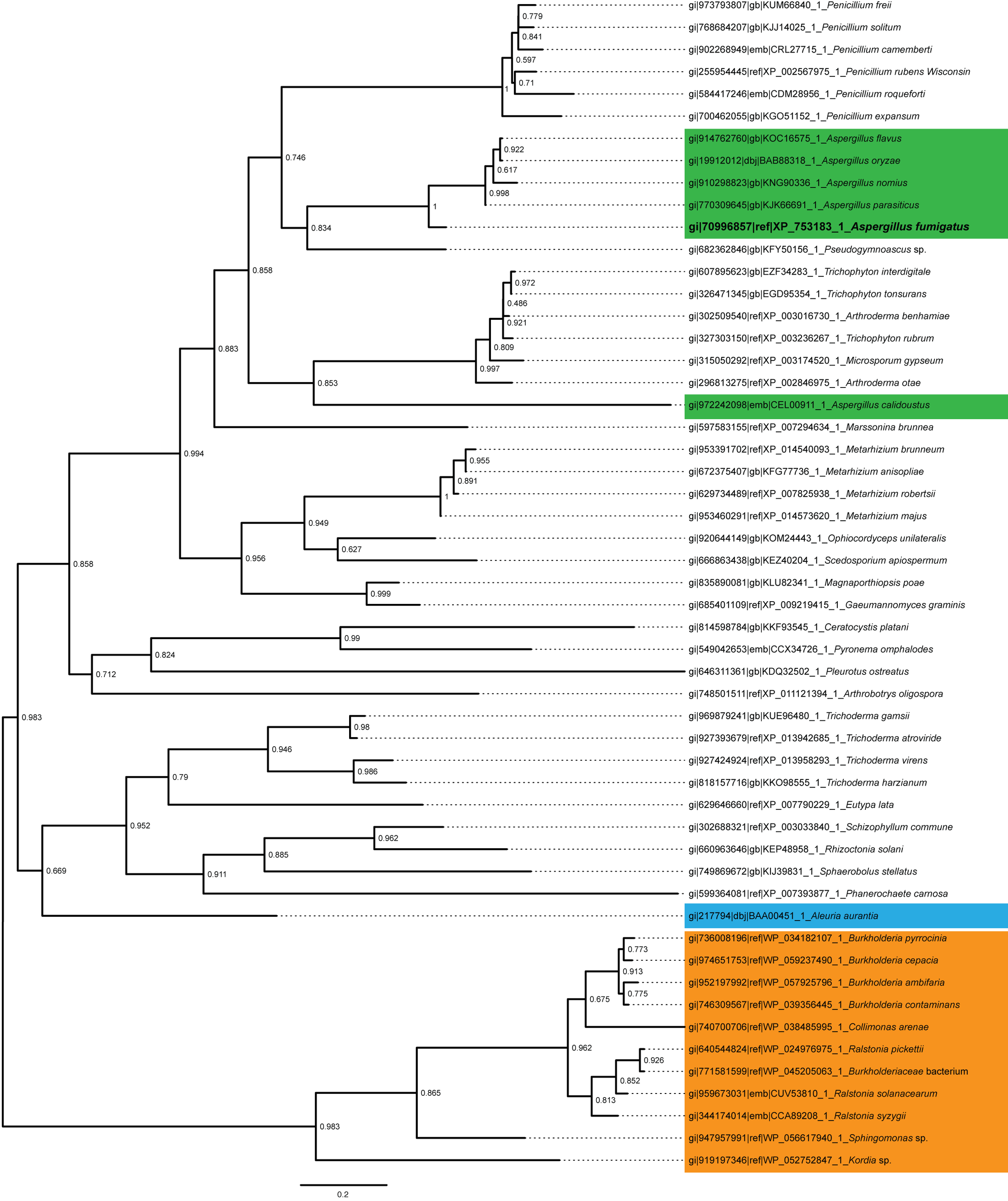

Supplement: S1 Fig — Aspergillus and bacterial fucose-binding lectins are highlighted in green and orange boxes, respectively. Aleuria aurantia lectin (AAL) was used to BLAST fungal and bacterial fucose-binding lectin protein sequences deposited in NCBI (blue box). A multiple sequence alignment of the selected sequences was used to identify the conserved region among bacterial and fungal sequences, which was extracted and subsequently used for phylogenetic analysis. Bootstrap values are presented at nodes. (TIF) [file ppat.1005555.s001.tif]

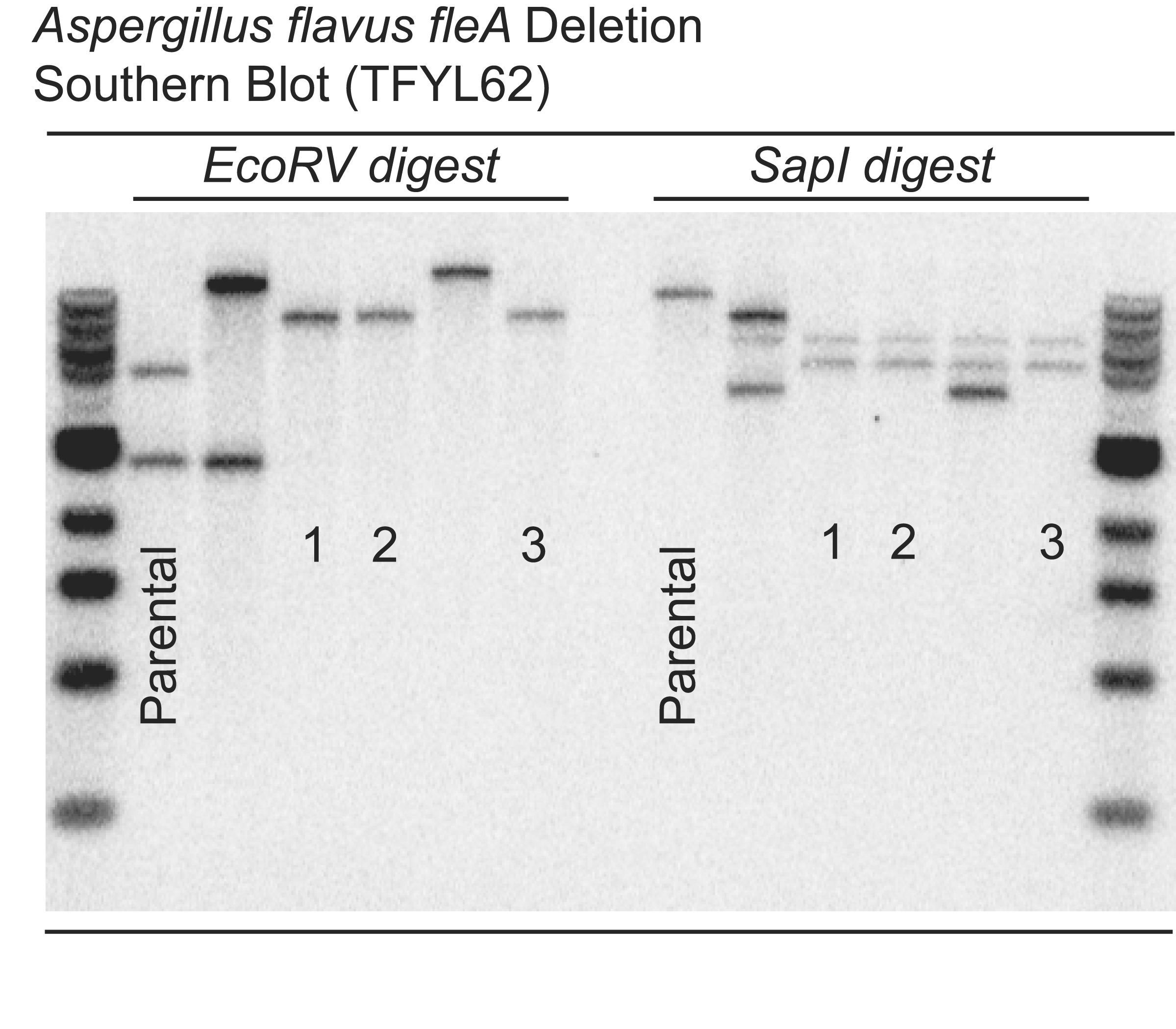

Supplement: S2 Fig — Southern blot depicting successful deletion of fleA in A. flavus (TIF) [file ppat.1005555.s002.tif]
