# Supplementary material for: FleA Expression in Aspergillus fumigatus Is Recognized by Fucosylated Structures on Mucins and Macrophages to Prevent Lung Infection
Source: PLoS Pathog. 2016 Apr 8;12(4):e1005555. doi: 10.1371/journal.ppat.1005555 (PMC4825926; doi:10.1371/journal.ppat.1005555)
Supplement: S1 Table — (PDF) [file ppat.1005555.s004.pdf]

**Supplementary Table 1**	**WT Concentration (pg/ml)**	**ΔfleA concentration (pg/ml)**	**p value**
**Cytokines**			
GM-CSF	27.6 ± 13.6	38.7 ± 16.1	0.27
IFN-γ	187.2 ± 91.6	118.1 ± 24.0	0.14
IL-1α	53.8 ± 13.24	66.7 ± 39.0	0.50
IL-1β	184.0 ± 27.9	181.6 ± 26.9	0.89
IL-2	93.5 ± 36.9	79.1 ± 8.4	0.42
IL-4	17.4 ± 2.6	20.1 ± 4.8	0.30
IL-5	93.5 ± 31.0	125.3 ± 95.0	0.50
IL-6	1444.86 ± 337.3	2571.2 ± 1242.4	0.09
IL-10	Below Range	Below Range	
IL-12	3330.1 ± 1338.4	2211.9 ± 839.2	0.15
IL-13	Below Range	Below Range	
IL-17	747.6 ± 541.7	495.8 ± 338.2	0.40
TNF-α	Above Range	Above Range	
**Chemokines**			
MIP-1α	4453.4 ±1354.3	9797.4 ± 8686.9	0.12
MCP-1	2581.7 ± 1065.8	7834.7 ± 4540.6	0.22
IP-10	15344.8 ± 21573.5	4375 .2 ± 4333.2	0.31
MIG	13497.9 ± 6266.1	6795.7 ± 5143.0	0.10
KC	Below Range	Below Range	0.14
**Growth Factors**			
FGF Basic	16.7 ± 5.2	19.8 ± 5.9	0.41
VEGF	2821.7 ± 552.5	3709.2 ± 38.9	0.09

Table S1. Luminex assay in BAL from WT and *ΔfleA* treated animals.
