# Supplementary material for: FleA Expression in Aspergillus fumigatus Is Recognized by Fucosylated Structures on Mucins and Macrophages to Prevent Lung Infection
Source: PLoS Pathog. 2016 Apr 8;12(4):e1005555. doi: 10.1371/journal.ppat.1005555 (PMC4825926; doi:10.1371/journal.ppat.1005555)
Supplement: S2 Table — (PDF) [file ppat.1005555.s005.pdf]

**Supplementary Table 2**	**Strains of *Aspergillus* used**	
**Fungal Strain**	**Genotype**	**Source or Reference**
	Aspergillus fumigatus	
AF293	Wild type	(1)
AF293.1	*pyrG1*	(2)
AF293.6	*pyrG1, argB1*	(1)
TJMP131.5	*pyrG1; AfpyrG::gpdA(p):GFP:H2A*	This study
TGJF5.3	*pyrG1, argB1; AfpyrG::gpdA(p)::GFP::H2A*	This study
TGJF6.7	*pyrG1, argB1; AfpyrG::gpdA(p)::GFP::H2A, ∆fleA::A. fumigatus argB*	This study
TGJF6.8	*pyrG1, argB1; AfpyrG::gpdA(p)::GFP::H2A, ∆fleA::A. fumigatus argB*	This study
TGJF6.13	*pyrG1, argB1; AfpyrG::gpdA(p)::GFP::H2A, ∆fleA::A. fumigatus argB*	This study
	Aspergillus flavus	
CA14	Wild type	(3)
∆ku70∆pyrG	*ΔpyrG::cypA; ΔnkuA*	(4)
TKJA13.1	*ΔpyrG::cypA; ΔnkuA::fumi pyrG*	(5)
TFYL62.1	*ΔfleA::fumi pyrG; ΔpyrG::cypA. ΔnkuA*	This study
TFYL62.2	*ΔfleA::fumi pyrG; ΔpyrG::cypA. ΔnkuA*	This study
TFYL62.3	*ΔfleA::fumi pyrG; ΔpyrG::cypA. ΔnkuA*	This study

1. Xue T, Nguyen CK, Romans A, Kontoyiannis DP, May GS. Isogenic auxotrophic mutant strains in the *Aspergillus fumigatus* genome reference strain

AF293. Archives of microbiology. 2004 Nov;182(5):346-53. PubMed PMID: 15365692.

2. Osherov N, Kontoyiannis DP, Romans A, May GS. Resistance to itraconazole in *Aspergillus nidulans* and *Aspergillus fumigatus* is conferred by extra copies of the *A. nidulans* P-450 14alpha-demethylase gene, pdmA. J Antimicrob Chemother. 2001 Jul;48(1):75-81. PubMed PMID: 11418514.

3. Hua SS, Tarun AS, Pandey SN, Chang L, Chang PK. Characterization of AFLAV, a Tf1/Sushi retrotransposon from *Aspergillus flavus*. Mycopathologia. 2007 Feb;163(2):97-104. PubMed PMID: 17286166.

4. Chang PK, Scharfenstein LL, Wei Q, Bhatnagar D. Development and refinement of a high-efficiency gene-targeting system for *Aspergillus flavus*. Journal of microbiological methods. 2010 Jun;81(3):240-6. PubMed PMID: 20298723.

5. Affeldt KJ, Carrig J, Amare M, Keller NP. Global survey of canonical *Aspergillus flavus* G protein-coupled receptors. mBio. 2014;5(5):e01501-14. PubMed PMID: 25316696. Pubmed Central PMCID: 4205791.
