# Supplementary material for: FleA Expression in Aspergillus fumigatus Is Recognized by Fucosylated Structures on Mucins and Macrophages to Prevent Lung Infection
Source: PLoS Pathog. 2016 Apr 8;12(4):e1005555. doi: 10.1371/journal.ppat.1005555 (PMC4825926; doi:10.1371/journal.ppat.1005555)
Supplement: S3 Table — (PDF) [file ppat.1005555.s006.pdf]

Supplemental Table 3. Primers used in this study.

Primer/Plasmid	Sequence	Purpose
GF FleA del F (P1)	**5'**-CATTAGGTGCATATGACGTG-**3'**	5' Flank
GF FleA del F (P2)	**5'**-GGCCTTAGAAGAGTCAGTC-**3'**	Cassette fusion
GF FleA del R (P3)	**5'**-GAAAATTTGTCTTGGATGCAGACCGCGTTCGGTCTTGCGGTATCTTGTCAG-**3'**	5' Flank
GF A. fumi argB F (P4)	**5'**-GAACGCGGTCTGCATCCAAG-**3'**	*argB*
GF A. fumi argB R (P5)	**5'**-GAAGGAGAGACCCATACATCC-**3'**	*argB*
GF FleA del F (P6)	**5'**-GATCAAATGGATGTATGGGTCTCTCCTTCGCTATAGACTACTACAGGTG-**3'**	3' Flank
GF FleA del R (P7)	**5'**-GGGAACATGTACGTGCTAG-**3'**	Cassette fusion
GF FleA del R (P8)	**5'**-GGATGAGATCCTCCTTGTAG-**3'**	3' Flank, 3' *fleA* RFP Flank
GF argB QC F (pGJF7)	**5'**-CGTTAAAAGTTCCGTGGAGTTACCAGTGATTGACCAGAACGCGGTCTGCATCCAAG-**3'**	pGJF7.2 Plasmid
GF argB QC R (pGJF6-7)	**5'**-GCCCTCTGTCTGAGAGGAGGCACTGATGCGAAGGAGAGACCCATACATCC-**3'**	pGJF7.2 Plasmid
GF FleA Native Tag P1 F	**5'**-GGAACAACCGTCTAGCTACATGC-**3'**	5' *fleA* RFP Flank
GF FleA Native Tag P3 R	**5'**-GGCACCGGCTCCAGCGCCTGCACCAGCTCCAGCAGGAGGAAGAGCACTTC-**3'**	5' *fleA* RFP Flank
GF FleA Native Tag P6 F	**5'**-TCCTTCGCATCAGTGCCTCCTCTCAGACAGGCTATAGACTACTACAGGTG-**3'**	3' *fleA* RFP Flank
GF/JP GFP/RFP F	**5'**-GGAGCTGGTGCAGGCGCTG -**3'**	RFP/*argB* cassette
GF/JP GFP/RFP R	**5'**-CTGTCTGAGAGGAGGCACTG-**3'**	RFP/*argB* cassette
GF FleA Native Tag P2 F	**5'**-GGCCTCAATGGCCTCTATGC-**3'**	RFP cassette fusion
JP AfpyrG ClaI For	**5'**-GAGAATATCGATCCTCAAACAATGCTCTT	*AfpyrG*

	C**-3’**	
JP AfpyrG ClaI Rev	**5’**-CCAGGTATCGATTCGGGAGGTATTG**-3’**	*AfpyrG*
pGJF7.2	**Plasmid**	RFP*:argB* tag
pJMP51	**Plasmid**	Histone GFP
FY AFLA FleA 5’ FOR	**5’**-GGTGGAGACTCGATACAGCTC**-3’**	5’ flank
FY AFLA FleA 5’ REV	**5’-** GAAGAGGGTGAAGAGCATTGTTTGAGGC AGCCGCAAGTGAAAGTTTCG**-3’**	5’ flank
FY AFLA FleA 3’ FOR	**5’-** GACGACAATACCTCCCGACGATACCTGG GGTCTGTCTACTACCGACCG**-3’**	3’ flank
FY AFLA FleA 3’ REV	**5’**-CGCCATTAACCCTCATCGCTG**-3’**	3’ flank
FY AFLA FleA Nest FOR	**5’**-GGCCAAGGTGCTCTAGCC**-3’**	Whole deletion cassette
FY AFLA FleA Nest REV	**5’**-CGGACACGATGGAGGGTCG**-3’**	Whole deletion cassette
KS Afu pyrG FOR	**5’**-TGCCTCAAACAATGCTCTTC**-3’**	*A. fumigatus pyrG*
KS Afu pyrG REV	**5’**-CCAGGTATCGTCGGGAGGT**-3’**	*A. fumigatus pyrG*
18S RNA forward	**5’**-ACGAGACCTCGGCCCTTA**-3’**	5’ flank
18S RNA reverse	**5’**-AGGGCATCACAGACCTGTT**-3’**	3’ flank
18S RNA probe	**5’**-CTATCGGCTCAAGCCGATGGAAG**-3’**	qPCR
HPRT forward	**5’**-AGGTTGCAAGCTTGCTGGT**-3’**	5’ flank
HRPT reverse	**5’**-TGAAGTACTCATTATAGTCAAGGGCA**-3’**	3’ flank
HPRT probe	**5’**-TGTTGGATACAGGCCAGACTTTGT**-3’**	qPCR
