# Supplementary material for: FleA Expression in Aspergillus fumigatus Is Recognized by Fucosylated Structures on Mucins and Macrophages to Prevent Lung Infection
Source: PLoS Pathog. 2016 Apr 8;12(4):e1005555. doi: 10.1371/journal.ppat.1005555 (PMC4825926; doi:10.1371/journal.ppat.1005555)
Supplement: S1 Text — (DOCX) [file ppat.1005555.s007.docx]

**S1 Text**

**FleA Expression in *Aspergillus fumigatus* is Recognized by Fucosylated Structures on Mucins and Macrophages to Prevent Lung infection.**

**Authors:** Sheena C. Kerr^1¶^, Gregory J. Fischer^2¶^, Meenal Sinha^3^, Orla McCabe^4^, Jonathan M. Palmer^5^, Tsokyi Choera^5^, Fang Yun Lim^5^, Michaela Wimmerova^6^, Stephen D. Carrington^7^, Shaopeng Yuan^1^, Clifford A. Lowell^3^, Stefan Oscarson^4^, Nancy P.Keller^5^ and John V. Fahy^1^*.

**Affiliations:** ^1^ Division of Pulmonary and Critical Care Medicine, University of California, San Francisco, CA 94143

^2^ Department of Genetics, University of Wisconsin, Madison, WI 53706

^3^ Department of Laboratory Medicine, University of California, San Francisco, CA 94143.

^4^ Center for Synthesis and Chemical Biology, University College Dublin, Ireland.

^5^ Department of Medical Microbiology and Immunology, University of Wisconsin, Madison, WI 53706.

^6^ Faculty of Science and Central European Institute of Technology, Masaryk University, Brno, CZ-62500, Czech Republic.

^7^ Veterinary Science Centre, School of Agriculture, Food Science and Veterinary Medicine, University College Dublin, Dublin, Ireland.

*Correspondence to: John V. Fahy

Email: john.fahy@ucsf.edu

^¶^These two authors contributed equally to this work

**Materials and Methods**

**Phylogenetic analysis of fucose-binding lectins using FastTree Neighbor-Joining method.**

Phylogenetic analysis of fucose-specific lectins was carried out based on previous homology searches to *Aleuria aurantia* lectin (AAL; protein sequence BAA00451 [[1](#_ENREF_1), [2](#_ENREF_2)]). The AAL protein sequence was used to identify fungal and bacterial sequences from the National Center for the Biotechnology Information (NCBI) database by performing a protein BLAST search. A sole bacterial fucose-binding lectin was identified in *Ralstonia solanacearum* (RSL; protein sequence CUV53810.1), which was used to subsequently BLAST bacterial sequences. Bacterial and fungal sequences with an e-value less than or equal to 1x10^-10^ were included in subsequent steps. An initial multiple sequence alignment of complete sequences was performed using MAFFT (http://mafft.cbrc.jp/alignment/software/) [[3](#_ENREF_3)] Using the AliView multiple sequence alignment editor [[4](#_ENREF_4)], the conserved region among bacterial and fungal sequences was extracted and run through FastTree (http://www.microbesonline.org/fasttree/) [[5](#_ENREF_5)] to compute a phylogenetic tree based on the Neighbor-Joining method. Results were visualized using FigTree (http://tree.bio.ed.ac.uk/soft- ware/figtree/) and midpoint rooted.

**Sputum Induction**

Sputum inductions were performed according to standard protocols [[6](#_ENREF_6)]. Briefly, subjects inhaled a nebulized solution of 3% saline through a mouthpiece for 20 minutes, pausing every 2 minutes to spit saliva into a saliva cup and sputum into a sputum cup. A 1ml aliquot of sputum was processed for total and differential cell counts by the addition of 10% Sputolysin (EMD Millipore, Billerica, MA) at a 1:1 g/ml (sputum weight: Sputolysin) ratio, mixing with a pipette and incubating at 37^o^C with agitation. Samples were removed for additional physical mixing every 5 minutes for a total of 15 minutes and used for measurement of cell differentials. Samples containing less than 50% squamous cells were approved for mucin purification. An equal volume of 8M guanidine hydrochloride was added to the remainder of the sputum and rotated at 4^o^C until homogenized.

**Purification of high molecular weight mucin from sputum**

High molecular weight mucin was purified from induced sputum from healthy subjects, as described previously [[7](#_ENREF_7)]. Briefly, sputum samples mixed with 8M GuHCl in a 1:1 ratio were subjected to a cesium chloride gradient with a density of 1.4 g/mL to separate the mucins from proteins, glycoproteins and nucleic acids. Carbohydrate-rich fractions as confirmed by slot blotting with PAS detection pertaining to 1.3-1.47 g/mL were then subjected to gel filtration on a Sepharose CL4B column in 50 mM Tris, 100mM KCl pH 7.5. Undegraded mucins identified by PAS staining on a slot blot were present in the Vo fraction of this column. This material was then desalted on Sephadex G25 prior to freeze drying and quantified by dried weight.

**Mucin plate- binding assay**

Recombinant FleA (prepared as [[1](#_ENREF_1)]) was incubated with 100 mM fucose in PBS for 1 hour then biotinylated with EZ-link sulfo NHS biotin (Pierce, Thermo Fisher, Rockford, IL) according to manufacturers instructions. Labeled protein was dialysed against TBS to remove fucose. Purified human mucin was coated on a Nunc maxisorp plate at 20 μg/ml in carbonate bicarbonate buffer pH 9.6 overnight at 4 ^o^C. After washing with TBS + 0.05% Tween-20, 10 mM CaCl_2_, 3% BSA, plates were blocked with the same buffer. Biotinylated recombinant FleA was incubated at 5 μg/ml in TBS + 0.05% Tween-20, 10 mM CaCl_2_, 1% BSA (binding buffer) in the presence or absence of 100 mM fucose or 100 mM galactose. For inhibition assays, recombinant FleA was incubated with a dilution series of synthesized carbohydrate compounds starting at 5 mM. Plates were washed three times with binding buffer and incubated with ExtrAvidin-alkaline phosphatase (Sigma-Aldrich, St Louis, MO) at 1:10000 in binding buffer. After three washes with binding buffer, the plates were detected using Phosphatase substrate (Sigma-Aldrich, St Louis, MO) in carbonate bicarbonate buffer pH 9.6 + 1 mM MgCl_2_ and read at 405nm in a Biotek Synergy plate reader (Biotek, Winooski, VT).

**Synthesis of carbohydrates**

General methods

Unless noted, chemical reagents and solvents were used without further purification from commercial sources. Reactions were magnetically stirred. Concentration *in vacuo* was generally performed using a Buchi rotary evaporator. The ^1^H / ^13^C NMR spectra (δ in ppm, relative to TMS in CDCl_3_) were recorded with Varian spectrometers (400/101 MHz or 500/125 MHz) at 25 °C. Assignments were aided by ^1^H-^1^H and ^1^H-^13^C correlation experiments. HRMS spectra were recorded on a micromass LCT instrument from Waters. Optical rotations were recorded on a Perkin-Elmer polarimeter (Model 343) at the sodium D-line (589 nm) at 20 °C using a 1 dm cell, and are not corrected. Silica gel chromatography was carried out using Davisil LC60A SiO_2_ (40-63 µm) silica gel. All reactions were monitored by thin-layer chromatography (TLC). TLC was performed on Merck DC-Alufolien plates precoated with silica gel 60 F254. They were visualized with UV-light (254 nm) fluorescence quenching, and/or by charring with an 8% H_2_SO_4_ dip (stock solution: 8 mL conc. H_2_SO_4_, 92 mL EtOH), and/or ninhydrin dip (stock solution: 0.3 g ninhydrin, 3 mL AcOH, 100 mL EtOH).

**Ethyl 2,3,4-tri-*O*-acetyl-1-thio-L-fucopyranoside (2)**

Under an atmosphere of nitrogen, L-fucose **1** (3.00 g, 18.3 mmol) was dissolved in dry pyridine (30 ml). Acetic anhydride (13.79 ml, 146.2 mmol) was added and the reaction was stirred at room temperature (RT) overnight. The solvent was removed under reduced pressure. The residue was diluted with CH_2_Cl_2_ (50 ml) and washed successively with 1M HCl, sat. aq. NaHCO_3,_ and water. The organic layer was dried over MgSO_4_, filtered and concentrated. The crude product was then purified by silica gel chromatography (toluene/ethyl acetate 2:1, v/v) to give 1,2,3,4-tetra-*O*-acetyl-L-fucopyranose (5.81 g, 96%, α:β = 1.5:1) as a clear syrup. **R_f_:** 0.91 (toluene/EtOAc, 2:1 v/v); ^1^H NMR (500 MHz, CDCl_3_) δ 6.34 (d, *J* = 3.0 Hz, 1H, H-1_α_), 5.68 (d, *J* = 8.3 Hz, 1H, H-1_β_), 5.37 – 5.29 (m, 3H, H-2, H-3, H-4), 4.30 – 4.24 (m, 1H, H-5), 2.18 (s, 3H, C*H*_3-OAc_), 2.15 (s, 3H, C*H*_3-OAc_), 2.01 (s, 3H, C*H*_3-OAc_), 2.00 (s, 3H, C*H*_3-OAc_), 1.16 (d, *J* = 6.5 Hz, 3H, C*H*_3_-6). 1,2,3,4-tetra-*O*-acetyl-L-fucopyranose (5.68 g, 17.1 mmol) was then dissolved in dry CH_2_Cl_2_ (60 ml) and 4Å molecular sieve were added under an atmosphere of nitrogen. The mixture was stirred at RT for 30 min and then cooled to 0 ˚C. Ethanethiol (1.5 ml, 20.5 mmol) and BF_3_·OEt_2_ (4.3 ml, 34.2 mmol) were added and the reaction was stirred at rt overnight. The reaction mixture was quenched with Et_3_N and filtered through Celite. It was then washed successively with 1M sulfuric acid, NaHCO_3_ and water. The mixture was dried over MgSO_4_, filtered and concentrated. The crude product was purified by silica gel chromatography (toluene/EtOAc 8:1, v/v) to afford **2** (5.10 g, 89%, α:β = 1:3) as a semi-transparent resin. R_f_: **2α** = 0.58, **2β** = 0.50 (toluene/EtOAc, 2:1 v/v); **2α:** ^1^H NMR (500 MHz, CDCl_3_) δ 5.70 (d, *J* = 5.4 Hz, 1H, H-1), 5.29 (dd, *J* = 3.1, 0.8 Hz, 1H, H-4), 5.26 (dd, *J* = 10.9, 5.4 Hz, 1H, H-2), 5.22 (dd, *J* = 10.8, 3.2 Hz, 1H, H-3), 4.49 (dt, *J* = 13.0, 6.4 Hz, 1H, H-5), 2.62 – 2.48 (m, 2H, -SC*H*_2_CH_3_), 2.16 (s, 3H, C*H*_3-OAc_), 2.07 (s, 3H, C*H*_3-OAc_), 1.99 (s, 3H, C*H*_3-OAc_), 1.26 (t, *J* = 7.4 Hz, 3H, -SCH_2_C*H*_3_), 1.16 (d, *J* = 6.5 Hz, 3H, C*H*_3_-6); **2β:** ^1^H NMR (500 MHz, CDCl_3_) δ 5.27 (dd, *J* = 3.4, 0.6 Hz, 1H, H-4), 5.22 (t, *J* = 10.0 Hz, 1H, H-2), 5.05 (dd, *J* = 10.0, 3.4 Hz, 1H, H-3), 4.46 (d, *J* = 9.9 Hz, 1H, H-1), 3.86 – 3.79 (m, 1H, H-5), 2.80 – 2.66 (m, 2H, -SC*H*_2_CH_3_), 2.17 (s, 3H, C*H*_3-OAc_), 2.06 (s, 3H, C*H*_3-OAc_), 1.98 (s, 3H, C*H*_3-OAc_), 1.28 (t, *J* = 7.5 Hz, 3H, -SCH_2_C*H*_3_), 1.22 (d, *J* = 6.4 Hz, 3H, C*H*_3_-6).

**Ethyl 2,3,4-tri-*O*-benzyl-1-thio-L-fucopyranoside (3)**

Compound **2** (4.04 g, 12.1 mmol) was dissolved in dry MeOH (100 ml) and cooled to 0 ˚C. NaOMe (100 mg, 1.9 mmol) was added and the contents were stirred at 0 ˚C to RT over 3 h. The reaction was monitored by TLC (R_f_: 0.17 EtOAc/toluene, 5:1 v/v). The reaction was quenched with Dowex-50WX8 acid resin, filtered and concentrated *in vacuo*. The crude compound (2.51 g, 12.0 mmol), along with BnBr (8.67 ml, 72.9 mmol), was dissolved in dry DMF (100 ml) under an atmosphere of nitrogen. A solution of 60% NaH (2.83 g, 84.2 mmol) in dry DMF (40 ml) was made up under an atmosphere of N_2_ at 0 ˚C. The BnBr/sugar solution was added drop wise to the NaH solution. The reaction was stirred at RT for 5 h. The reaction was quenched by addition of methanol (30 ml). The mixture was diluted with toluene and washed with sat. aq. NaHCO_3,_ and water. The organic layer was dried over MgSO_4_, filtered and concentrated *in vacuo*. The crude product was purified by silica gel chromatography (toluene/EtOAc 19:1, v/v) to give **3** (5.46 g, 95%, α:β = 1:3) as a semi-transparent resin. R_f_: 0.56 (toluene/EtOAc 8:1, v/v); **3α:** ^1^H NMR (500 MHz, CDCl_3_) δ 7.60 – 7.14 (m, 15H, H-Ar), 5.46 (d, *J* = 5.5 Hz, 1H, H-1), 4.98 (d, *J* = 11.6 Hz, 1H, CH*H*Ph), 4.88 – 4.83 (m, 1H, CH*H*Ph), 4.77 – 4.61 (m, 4H, C*H*_2_Ph, C*H*_2_Ph), 4.28 (dd, *J* = 9.9, 5.5 Hz, 1H, H-2), 4.19 (q, *J* = 6.5 Hz, 1H, H-5), 3.78 (dd, *J* = 9.9, 2.9 Hz, 1H, H-3), 3.63 (d, *J* = 1.9 Hz, 1H, H-4), 2.61 – 2.43 (m, 2H, -SC*H*_2_CH_3_), 1.26 (t, *J* = 7.4 Hz, 3H, -SCH_2_C*H*_3_), 1.13 (d, *J* = 6.5 Hz, 3H, C*H*_3_-6); **3β:** ^1^H NMR (500 MHz, CDCl_3_) δ 7.41 – 7.23 (m, 15H, H-Ar), 5.01 – 4.97 (m, 1H, CH*H*Ph), 4.89 (d, *J* = 10.2 Hz, 1H, CH*H*Ph), 4.80 (d, *J* = 10.2 Hz, 1H, CH*H*Ph), 4.78 – 4.70 (m, 2H, C*H*_2_Ph), 4.70 – 4.67 (m, 1H, CH*H*Ph), 4.38 (d, *J* = 9.6 Hz, 1H, H-1), 3.82 (t, *J* = 9.4 Hz, 1H, H-2), 3.60 (d, *J* = 2.5 Hz, 1H, H-4), 3.55 (dd, *J* = 9.3, 2.8 Hz, 1H, H-3), 3.47 (q, *J* = 6.3 Hz, 1H, H-5), 2.81 – 2.66 (m, 1H, -SC*H*_2_CH_3_), 1.29 (t, *J* = 7.4 Hz, 3H, -SCH_2_C*H*_3_), 1.20 (d, *J* = 6.4 Hz, 3H, C*H*_3_-6).

L-Fucose

Scheme 1: (i) (a) Ac_2_O, dry Pyridine, RT, overnight, 96%, (b) EtSH, BF_3_·OEt_2_, dry CH_2_Cl_2_, overnight, 89%; (ii) (a) NaOMe, MeOH, 0 °C to RT, 3 h, (b) BnBr, NaH, dry DMF, RT, 5 h, 95%.

**2-Azidoethyl 2-*O*-benzyl-4,6-*O*-benzylidene-β-D-glucopyranoside (5)** and **2-Azidoethyl 3-*O*-benzyl-4,6-*O*-benzylidene-β-D-glucopyranoside (6)**

Compound **4** (1.0 g, 2.97 mmol), Bu_4_N(HSO_4_) (201 mg, 0.59 mmol) and BnBr (0.60 ml, 5.1 mmol) were dissolved in CH_2_Cl_2_ (60 ml). 5% aq. NaOH solution (5 ml) was added and the mixture was refluxed at 50 ˚C. After 48 h the reaction was cooled. The organic layer was separated from the aqueous layer and washed with water. It was then dried over MgSO_4_, filtered and concentrated. The crude was purified by silica gel chromatography (toluene/EtOAc 10:1 to 9:1, v/v) to give **5** (663 mg, 52%) and **6** (261 mg, 21%) both as white foams.

**5:** R_f_: 0.68 (toluene/EtOAc 2:1, v/v); ^1^H NMR (500 MHz, CDCl_3_) δ 7.48 (dd, *J* = 7.2, 2.2 Hz, 2H, H-Ar), 7.41 – 7.30 (m, 8H, H-Ar), 5.52 (s, 1H, C*H*Ph), 4.96 (d, *J* = 11.5 Hz, 1H, CH*H*Ph), 4.76 (d, *J* = 11.5 Hz, 1H, CH*H*Ph), 4.55 (d, *J* = 7.7 Hz, 1H, H-1), 4.34 (dd, *J* = 10.5, 5.0 Hz, 1H, H-6a), 4.07 – 4.02 (m, 1H, OCH*H*CH_2_N_3_), 3.85 (td, *J* = 9.2, 2.2 Hz, 1H, H-3), 3.81 – 3.72 (m, 2H, H-6b, -OCH*H*CH_2_N_3_), 3.55 (t, *J* = 9.4 Hz, 1H, H-4), 3.52 – 3.41 (m, 3H, -OCH_2_C*H*_2_N_3_, H-5 ), 3.38 (dd, *J* = 8.7, 7.8 Hz, 1H, H-2), 2.46 (d, *J* = 2.2 Hz, 1H, O*H*-3); ^13^C NMR (126 MHz, CDCl_3_) δ 138.22, 136.94 (2Cs, Cq-Ar), 129.19 - 126.24 (10Cs, C-Ar ), 103.79 (C-1), 101.79 (*C*HPh), 81.90 (C-2), 80.31 (C-4), 74.91 (*C*H_2_Ph), 73.19 (C-3), 68.65 (-O*C*H_2_CH_2_N_3_), 68.49 (C-6), 66.19 (C-5), 50.99 (-OCH_2_*C*H_2_N_3_). HRMS(ES^+^): calcd. for C_22_H_25_N_3_O_6_(Na^+^) requires 450.1641: found 450.1659. [α]_D_ = -27.5 (c 1.15, CHCl_3_).

**6:** R_f_: 0.5 (toluene/EtOAc, 2:1 v/v); ^1^H NMR (500 MHz, CDCl_3_) δ 7.52 – 7.45 (m, 2H, H-Ar), 7.41 – 7.26 (m, 8H, H-Ar), 5.57 (s, 1H, C*H*Ph), 4.96 (d, *J* = 11.7 Hz, 1H, CH*H*Ph), 4.81 (d, *J* = 11.7 Hz, 1H, CH*H*Ph), 4.45 (d, *J* = 7.6 Hz, 1H, H-1), 4.35 (dd, *J* = 10.5, 5.0 Hz, 1H, H-6a), 4.10 – 4.01 (m, 1H, -OCH*H*CH_2_N_3_), 3.79 (m, 2H, H-6b,-OCH*H*CH_2_N_3_), 3.71 (t, *J* = 9.3 Hz, 1H, H-3), 3.67 (m, 1H, H-4), 3.60 (dd, *J* = 11.9, 4.2 Hz, 1H, H-2), 3.55 – 3.49 (m, 1H, OCH_2_CH*H*N_3_), 3.49 – 3.43 (m, 1H, H-5), 3.43 – 3.36 (m, 1H, OCH_2_CH*H*N_3_), 2.50 (d, *J* = 2.5 Hz, 1H, O*H*-2); HRMS(ES^+^): calcd. for C_22_H_25_N_3_O_6_(Na^+^) requires 450.1641: found 450.1661; [α]_D_ = -27.4 (c 0.44, CHCl_3_)

**2-Azidoethyl 2,3-di-*O*-benzyl-4,6-*O*-benzylidene-β-D-glucopyranoside (7)**

A solution of NaH (380 mg, 15.8 mmol) in dry DMF (15 ml) was made up at 0 ˚C. Compound **4** (640 mg, 1.90 mmol) and BnBr (0.90 ml, 7.6 mmol) were dissolved in dry DMF (40 ml) and added drop wise to the NaH soln. The reaction was stirred at RT overnight. The reaction was quenched by addition of MeOH (10 ml). The mixture was diluted with toluene and washed with sat. aq. NaHCO_3,_ and water. The organic layer was dried over MgSO_4_, filtered and concentrated. The crude was purified by silica gel chromatography (toluene/EtOAc 13:1, v/v) to afford compound **7** (821 mg, 84%) as a white foam. R_f_: 0.77 (toluene/EtOAc 6:1, v/v); ^1^H NMR (500 MHz, CDCl_3_) δ 7.48 (dd, *J* = 7.6, 1.7 Hz, 2H, H-Ar), 7.40 – 7.26 (m, 13H, H-Ar), 5.57 (s, 1H, C*H*Ph), 4.91 (dd, *J* = 11.2, 2.5 Hz, 2H, CH*H*Ph-2,CH*H*Ph-3), 4.79 (dd, *J* = 11.2, 3.7 Hz, 2H, CH*H*Ph-2, CH*H*Ph-3), 4.54 (d, *J* = 7.7 Hz, 1H, H-1), 4.35 (dd, *J* = 10.5, 5.0 Hz, 1H, H-6a), 4.06 – 4.01 (m, 1H, -OC*H*_2_CH_2_N_3_), 3.79 (t, *J* = 9.2 Hz, 1H, H-6b), 3.77 – 3.72 (m, 2H, -OCH*H*CH_2_N_3_, H-3), 3.70 (t, *J* = 9.3 Hz, 1H, H-4), 3.55 – 3.38 (m, 4H, H-2, H-5, -OCH_2_C*H*_2_N_3_).

**2-Azidoethyl 2,3,6-tri-*O*-benzyl-β-D-glucopyranoside (8)**

Compound **7** (350 mg, 0.68 mmol), NaBH_3_CN (425 mg, 6.76 mmol) and 3Å MS were treated with dry THF (10 ml) and stirred at RT under an atmosphere of Ar. After 30 min, HCl solution (2M in diethyl ether) was added until a pH of 1 was attained. The reaction was closely monitored by TLC and after 4 h the mixture was filtered through Celite and concentrated. The crude product was purified by silica gel chromatography (toluene/EtOAc 5:1, v/v) to afford **8** (213 mg, 61%) as a white foam. Rf: 0.56 (toluene/EtOAc 3:1, v/v); ^1^H NMR (500 MHz, CDCl_3_) δ 7.38 – 7.24 (m, 15H, H-Ar), 4.95 (d, *J* = 11.1 Hz, 1H, CH*H*Ph), 4.92 (d, *J* = 11.5 Hz, 1H, CH*H*Ph), 4.74 – 4.71 (m, 2H, CH*H*Ph, CH*H*Ph), 4.58 (d, *J* = 12.2 Hz, 1H, CH*H*Ph), 4.55 (d, *J* = 12.0 Hz, 1H, CH*H*Ph), 4.43 (d, *J* = 7.4 Hz, 1H, H-1), 4.02 (ddd, *J* = 10.4, 5.6, 3.9 Hz, 1H, H-6a), 3.75 (dd, *J* = 10.5, 3.6 Hz, 1H, -OCH*H*CH_2_N_3_), 3.72 – 3.65 (m, 2H, H-6b, -OCH*H*CH_2_N_3_), 3.59 – 3.56 (m, 1H, H-4), 3.50 – 3.36 (m, 5H, H-2, H-3, H-5, -OCH_2_C*H*_2_N_3_), 2.41 (s, 1H, O*H*-4); ^13^C NMR (126 MHz, CDCl_3_) δ 138.63 - 137.96 (3Cs, Cq-Ar), 128.56 - 127.72 (15Cs, C-Ar), 103.75 (C-1), 84.02 (C-2), 81.77 (C-3), 75.27 (*C*H_2_Ph), 74.83 (*C*H_2_Ph), 74.31 (C-5), 73.70 (*C*H_2_Ph), 71.32 (C-4), 70.18 (-O*C*H_2_CH_2_N_3_), 68.19 (C-6), 51.07 (-OCH_2_*C*H_2_N_3_).

D-Glucose

**Scheme 2:** (i) Bu_4_N(HSO_4_), BnBr, NaOH, dry CH_2_Cl_2_, 50 ˚C, 40 h, 73% (**5**:**6** = 1.4:1)**;** (ii) BnBr, NaH, dry DMF, 0 ˚C to RT, 24 h, 84%**;** (iii) NaCNBH_3_, dry THF, HCl/Et_2_O, RT, 4 h, 61%.

**2-Azidoethyl 2,3,4-tri-*O*-benzyl-α-L-fucopyranosyl-(1→2)-3-*O*-benzyl-4,6-*O*-benzylidene-β-D-glucopyranoside (9)**

Donor **3** (551 mg, 1.15 mmol) was dissolved in dry CH_2_Cl_2_ (1 ml) under a N_2_ atmosphere and the mixture was cooled to 0 ˚C. Br_2_ (60 µL, 1.15 mmol) was added and the reaction was stirred while slowly attaining RT (~2 h). The reaction was cooled to 0 ˚C and quenched with cyclohexene. After co-evaporation with toluene, the residue was dissolved in dry CH_2_Cl_2_ (1 ml) under a N_2_ atmosphere. A mixture of acceptor **6** (164 mg, 0.38 mmol), Bu_4_NBr (371 mg, 1.15 mmol) and 4Å MS in dry CH_2_Cl_2_/DMF (1.8 ml, 6:1) was stirred at RT for 30 min under N_2_. The donor solution was added drop wise to the suspension and the reaction was stirred at RT overnight. The reaction was cooled to 0 ˚C and quenched with Et_3_N. The mixture was filtered through Celite, diluted with water and extracted with EtOAc. The organic phase was dried over MgSO_4_, filtered and concentrated. Purification by silica gel chromatography (cyclohexane/EtOAc 6:1, v/v) afforded disaccharide **9** (184 mg, 57%). R_f_: 0.6 (toluene/EtOAc 4:1, v/v); ^1^H NMR (500 MHz, CDCl_3_) δ 7.45 – 7.19 (m, 25H, H-Ar), 5.66 (d, *J* = 3.7 Hz, 1H, H-1_Fuc_), 5.52 (s, 1H, C*H*Ph), 5.02 (d, *J* = 11.5 Hz, 1H, CH*H*Ph), 4.95 (d, *J* = 11.4 Hz, 1H, CH*H*Ph), 4.82 (d, *J* = 11.7 Hz, 1H, CH*H*Ph), 4.75 – 4.70 (m, 2H, C*H*_2_Ph), 4.65 (d, *J* = 11.6 Hz, 1H, CH*H*Ph), 4.61 – 4.58 (m, 3H, C*H*_2_Ph, H-1_Glc_), 4.34 (dd, *J* = 10.6, 5.0 Hz, 2H, H-5_Fuc_, H-6a_Glc_), 4.05 (dd, *J* = 10.2, 3.7 Hz, 1H, H-2_Fuc_), 3.97 (ddd, *J* = 8.9, 7.6, 2.9 Hz, 3H, H-3_Fuc_, H-4_Glc_, -OCH*H*CH_2_N_3_), 3.90 (t, *J* = 7.8 Hz, 1H, H-2_Glc_), 3.75 (dt, *J* = 11.7, 9.7 Hz, 2H, H-6b_Glc_, H-3_Glc_), 3.71 (d, *J* = 1.4 Hz, 1H, H-4_Fuc_), 3.64 (ddd, *J* = 11.0, 7.5, 3.8 Hz, 1H, -OCH*H*CH_2_N_3_), 3.48 – 3.41 (m, 2H, H-5_Glc_, -OCH_2_CH*H*N_3_), 3.41 – 3.34 (m, 1H, -OCH_2_CH*H*N_3_), 1.14 (d, *J* = 6.5 Hz, 3H, C*H*_3_-6_Fuc_); ^13^C NMR (126 MHz, CDCl_3_) δ 138.92 -137.21 (5C, C-q Ar), 128.97-125.98 (25C, C-Ar), 102.37 (C-1_Glc_), 101.18 (*C*HPh), 97.38 (C-1_Fuc_), 82.44 (C-3_Fuc_), 81.78 (C-3_Glc_), 79.51 (C-4_Glc_), 77.92 (C-4_Fuc_), 75.86 (C-2_Fuc_), 75.19 (C-2_Glc_), 74.87 (*C*H_2_Ph), 74.27 (*C*H_2_Ph), 73.11 (*C*H_2_Ph), 73.02 (*C*H_2_Ph), 68.75 (C-6_Glc_), 67.68 (-O*C*H_2_CH_2_N_3_), 66.53 (C-5_Fuc_), 65.91 (C-5_Glc_), 50.86 (-OCH_2_*C*H_2_N_3_), 16.60 (C-6_Fuc_).

**2-Azidoethyl 2,3,4-tri-*O*-benzyl-α-L-fucopyranosyl-(1→3)-2-*O*-benzyl-4,6-*O*-benzylidene -β-D-glucopyranoside (10)**

Donor **3** (797 mg, 1.67 mmol) was dissolved in dry CH_2_Cl_2_ (10 ml) under a N_2_ atmosphere and the mixture was cooled to 0 ˚C. Br_2_ (108 µL, 2.11 mmol) was added and the reaction was stirred at RT for 1.5 h. The reaction was cooled to 0 ˚C and quenched with cyclohexene. After co-evaporation with toluene, the residue was dissolved in dry CH_2_Cl_2_ (2 ml) under a N_2_ atmosphere. The solution was added to a mixture of acceptor **5** (300 mg, 0.70 mmol), Et_4_NBr (443 mg, 2.11 mmol) and 4Å MS in dry CH_2_Cl_2_/DMF (4:1, 7 ml) and the reaction was stirred overnight at RT. The mixture was quenched with Et_3_N, filtered through Celite, diluted with water and extracted with CH_2_Cl_2_. The organic phase was dried over MgSO_4_, filtered and concentrated. The crude product was purified by silica gel chromatography (cyclohexane/EtOAc 7:1, v/v) yielding disaccharide **10** (378 mg, 64%). R_f_: 0.38 (toluene/EtOAc, 6:1 v/v); ^1^H NMR (500 MHz, CDCl_3_) δ 7.49 – 6.98 (m, 25H, H-Ar), 5.50 (d, *J* = 3.6 Hz, 1H, H-1_Fuc_), 5.41 (s, 1H, C*H*Ph), 4.88 (d, *J* = 11.6 Hz, 1H, CH*H*Ph), 4.80 (d, *J* = 11.5 Hz, 1H, CH*H*Ph), 4.71 (d, *J* = 11.6 Hz, 1H, CH*H*Ph), 4.63 (dd, *J* = 18.0, 11.6 Hz, 2H, C*H*_2_Ph), 4.54 (d, *J* = 11.8 Hz, 1H, CH*H*Ph), 4.48 (dd, *J* = 11.5, 5.2 Hz, 2H C*H*_2_Ph), 4.46 (d, *J* = 6.18 Hz, 1H, H-1_Glc_), 4.25 (dd, *J* = 10.5, 4.9 Hz, 1H, H-6a_Glc_), 4.14 (d, *J* = 6.5 Hz, 1H, H-5_Fuc_), 4.04 (t, *J* = 9.2 Hz, 1H, H-3_Glc_), 3.93 (dd, *J* = 10.2, 3.6 Hz, 1H, H-2_Fuc_), 3.91 – 3.86 (m, 2H, H-3_Fuc_, -OCH*H*CH_2_N_3_), 3.68 (t, *J* = 10.3 Hz, 1H, H-6b_Glc_), 3.61 (t, *J* = 9.5 Hz, 1H, H-4_Glc_), 3.59 – 3.54 (m, 2H, H-2_Glc_, -OCH*H*CH_2_N_3_), 3.42 (d, *J* = 1.2 Hz, 1H, H-4_Fuc_), 3.37 (td, *J* = 9.8, 5.0 Hz, 1H, H-5_Glc_), 3.28 (ddd, *J* = 10.3, 6.3, 4.1 Hz, 2H, -OCH_2_C*H*_2_N_3_), 0.75 (d, *J* = 6.4 Hz, 3H, C*H*_3_-6_Fuc_); ^13^C NMR (126 MHz, CDCl_3_) δ 138.86 - 137.38 (5C, Cq-Ar), 129.10 - 127.21 (25C, C-Ar), 104.26 (C-1_Glc_), 101.73 (*C*HPh), 97.55 (C-1_Fuc_), 83.49 (C-2_Glc_), 79.70 (C-3_Fuc_), 79.20 (C-4_Glc_), 77.96 (C-4_Fuc_), 75.64 (C-2_Fuc_), 74.89 (C-3_Glc_), 74.88 (*C*H_2_Ph), 74.14 (*C*H_2_Ph), 73.25 (*C*H_2_Ph), 72.90 (*C*H_2_Ph), 68.84 (C-6_Glc_), 68.46 (-O*C*H_2_CH_2_N_3_), 66.38 (C-5_Glc_), 66.18 (C-5_Fuc_), 50.92 (-OCH_2_*C*H_2_N_3_), 16.32 (C-6_Fuc_).

**2-Azidoethyl 2,3,4-tri-*O*-benzyl-α-L-fucopyranosyl-(1→4)-2,3,6-tri-*O*-benz-yl-β-D-gluco-pyranoside (11)**

Donor **3** (365 mg, 0.76 mmol) was dissolved in dry CH_2_Cl_2_ (2 ml) under a N_2_ atmosphere and the mixture was cooled to 0 ˚C. Br_2_ (52µL, 1.02 mmol) was added and the reaction was stirred while slowly attaining RT (~2 h). The reaction was cooled to 0 ˚C and quenched with cyclohexene. After co-evaporation with toluene, the residue was dissolved in dry CH_2_Cl_2_ (1 ml) under a N_2_ atmosphere. A mixture of acceptor **8** (132 mg, 0.25 mmol), Bu_4_NBr (246 mg, 0.76 mmol) and 4Å MS in dry CH_2_Cl_2_/DMF (6:1, 1.8 ml) was stirred at RT for 30 min under N_2_. The donor solution was added drop wise to the suspension and the reaction was stirred overnight. The reaction was cooled to 0 ˚C and quenched with Et_3_N. The mixture was filtered through Celite, diluted with water and extracted with EtOAc. The organic phase was dried over MgSO_4_, filtered and concentrated. Silica gel chromatography (cyclohexane/EtOAc, 7:1 → 6:1 v/v) was carried out to obtain disaccharide **11** (157 mg, 66%). R_f_: 0.52 (toluene/EtOAc, 6:1); ^1^H NMR (500 MHz, CDCl_3_) δ 7.41 – 7.18 (m, 30H, H-Ar), 5.08 (d, *J* = 3.6 Hz, 1H, H-1_Fuc_), 4.98 (d, *J* = 10.9 Hz, 1H, CH*H*Ph), 4.93 (d, *J* = 11.0 Hz, 1H, CH*H*Ph), 4.88 (d, *J* = 11.6 Hz, 1H, CH*H*Ph), 4.77 (dd, *J* = 11.7, 3.1 Hz, 2H, C*H*_2_Ph), 4.68 (dd, *J* = 11.4, 3.7 Hz, 2H, C*H*_2_Ph), 4.61 – 4.56 (m, 1H, CH*H*Ph), 4.58 - 4.54 (m, 2H, C*H*_2_Ph), 4.42 (m, 3H, C*H*_2_Ph, H-1_Glc_), 4.04 – 3.95 (m, 3H, CH*H*CH_2_N_3_, H-2_Fuc_, H-5_Fuc_), 3.84 – 3.78 (m, 2H, H-3_Fuc_, H-4_Glc_), 3.77 (dd, *J* = 8.9, 1.8 Hz, 1H, H-6a_Glc_), 3.72 – 3.67 (m, 1H, -OCH*H*CH_2_N_3_), 3.65 (dd, *J* = 11.0, 4.9 Hz, 1H, H-6b_Glc_), 3.59 (t, *J* = 9.0 Hz, 1H, H-3_Glc_), 3.48 (ddd, *J* = 12.0, 11.1, 5.7 Hz, 3H, H-2_Glc_, H-5_Glc_, -OCH_2_CH*H*N_3_), 3.40 (ddd, *J* = 13.0, 5.4, 4.1 Hz, 1H, -OCH_2_CH*H*N_3_), 3.35 (d, *J* = 1.4 Hz, 1H, H-4_Fuc_), 0.71 (d, *J* = 6.5 Hz, 3H, C*H*_3_-6_Fuc_); ^13^C NMR (126 MHz, CDCl_3_) δ 138.79 - 138.27 (6C, Cq-Ar), 128.38 - 127.37 (30C, C-Ar), 103.66 (C-1_Glc_), 97.63 (C-1_Fuc_), 82.99 (C-3_Glc_), 82.70 (C-2_Glc_), 79.40 (C-3_Fuc_), 77.61 (C-4_Fuc_), 76.43 (C-2_Fuc_), 75.45 (*C*H_2_Ph), 75.31 (C-5_Glc_), 74.75 (*C*H_2_Ph), 74.72 (*C*H_2_Ph), 74.25 (*C*H_2_Ph), 73.94 (C-4_Glc_), 73.24 (*C*H_2_Ph), 72.74 (*C*H_2_Ph), 68.80 (C-6_Glc_), 67.99 (-O*C*H_2_CH_2_N_3_), 66.78 (C-5_Fuc_), 51.05 (-OCH_2_*C*H_2_N_3_), 16.26 (C-6_Fuc_).

**General Procedure for Hydrogenolysis**

Pd/C 10 mol% (11.50 mmol) was added to a solution of the protected disaccharide (1.00 mmol) in THF/EtOH/H_2_O/1M HCl (5 ml, 20:20:1:1). Under an atmosphere of hydrogen (5 bar), the reaction was stirred at RT overnight. Upon completion, the mixture was filtered through a series of filters (20μm, 10μm, 5μm) and concentrated. The crude product was purified by size exclusion chromatography (P2 biogel) to give the deprotected disaccharide.

**2-Aminoethyl α-L-fucopyranosyl-(1→2)-β-D-glucopyranoside (12)**

^1^H NMR (500 MHz, D_2_O) δ 5.14 (d, *J* = 4.0 Hz, 1H, H-1_Fuc_), 4.55 (d, *J* = 7.8 Hz, 1H, H-1_Glc_), 1.14 (d, *J* = 6.6 Hz, 3H, C*H*_3_-6_Fuc_); ^13^C NMR (126 MHz, D_2_O) δ 101.33 (C-1_Glc_), 99.87 (C-1_Fuc_), 15.36 (C-6_Fuc_).

**2-Aminoethyl α-L-fucopyranosyl-(1→3)-β-D-glucopyranoside (13)**

^1^H NMR (500 MHz, D_2_O) δ 5.21 (d, *J* = 4.0 Hz, 1H, H-1_Fuc_), 4.46 (d, *J* = 8.0 Hz, 1H, H-1_Glc_), 1.12 (d, *J* = 6.6 Hz, 3H, C*H*_3_-6_Fuc_);

**2-Aminoethyl α-L-fucopyranosyl-(1→4)-β-D-glucopyranoside (14)**

^1^H NMR (500 MHz, D_2_O) δ 4.88 (d, *J* = 3.8 Hz, 1H, H-1_Fuc_), 4.45 (d, *J* = 8.0 Hz, 1H, H-1_Glc_), 1.12 (d, *J* = 6.6 Hz, 3H, C*H*_3_-6_Fuc_); ^13^C NMR (126 MHz, D_2_O) δ 102.01 (C-1_Glc_), 99.56 (C-1_Fuc_), 15.20 (C-6_Fuc_).

Disaccharides

Scheme 3: (i) (a) Br_2_, dry CH_2_Cl_2_, 0 ˚C → RT, 2h, (b) Bu_4_NBr or Et_4_NBr, 4Å MS, dry CH_2_Cl_2_/DMF, RT overnight.; (ii) H_2_, Pd/C, THF, RT, overnight.

**Allyl L-fucoside (15)**

L-Fucose **1** (5.0 g, 30.5 mmol) was suspended in allyl alcohol (16 ml). MeOH washed Dowex-50WX8 acid resin (3.0 g) was added and the reaction mixture was refluxed at 70 ˚C overnight. The mixture was filtered while hot and the filtrate was washed with MeOH. The solvent was evaporated and the α/β mixture was separated by silica gel chromatography (CH_2_Cl_2_/MeOH 6:1, v/v) to yield **15** (3.12 g, 50%) as a white solid. R_f_: 0.58 (CH_2_Cl_2_/MeOH 4:1, v/v); ^1^H NMR (400 MHz, CD_3_OD) δ 5.94 (ddd, *J* = 16.8, 10.9, 5.7 Hz, 1H, -OCH_2_C*H*CH_2_), 5.36 – 5.24 (m, 1H, -OCH_2_CHCH*H*), 5.14 (dd, *J* = 10.4, 1.7 Hz, 1H, -OCH_2_CHCH*H*), 4.77 (d, *J* = 3.0 Hz, 1H, H-1), 4.14 (ddt, *J* = 13.1, 5.2, 1.5 Hz, 1H, -OCH*H*CHCH_2_), 4.04 – 3.97 (m, 1H, -OCH*H*CHCH_2_), 3.93 (q, *J* = 6.7 Hz, 1H, H-5), 3.76 – 3.69 (m, 2H, H-2, H-3), 3.65 – 3.62 (m, 1H, H-4), 1.19 (d, *J* = 6.6 Hz, 3H, C*H*_3_-6).

**Allyl 2,3,4-tri-*O*-acetyl-L-fucoside (17)**

Compound **15** (250 mg, 1.2 mmol) was dissolved in dry pyridine (2 ml). Acetic anhydride (0.7 ml, 7.4 mmol) was added and the reaction was stirred at RT overnight. Upon completion, the solvent was removed under reduced pressure. The residue was dissolved in CH_2_Cl_2_, and washed successively with 1M HCl, sat. aq. NaHCO_3_, and H_2_O. The organic layer was then dried over MgSO_4_, filtered and concentrated. The crude product was purified by silica gel chromatography (toluene/EtOAc 7:1, v/v) to give **17** (367 mg, 91%) as a white solid. R_f_: α = 0.69, β = 0.56 (toluene/EtOAc 2:1, v/v); ^1^H NMR (500 MHz, CDCl_3_) δ 5.92 – 5.83 (m, 1H, -OCH_2_C*H*CH_2_), 5.39 (dd, *J* = 10.8, 3.4 Hz, 1H, H-3), 5.30 (m, 2H, H-4, -OCH_2_CHCH*H*), 5.22 – 5.19 (m, 1H, -OCH_2_CHCH*H*), 5.14 (dd, *J* = 10.8, 3.7 Hz, 1H, H-2), 5.09 (d, *J* = 3.7 Hz, 1H, H-1), 4.20 – 4.16 (m, 2H, H-5, -OCH*H*CHCH_2_), 4.01 (ddt, *J* = 13.1, 6.0, 1.3 Hz, 1H, -OCH*H*CHCH_2_), 2.16 (s, 3H, C*H*_3_-Ac), 2.07 (s, 3H, C*H*_3_-Ac), 1.98 (s, 3H, C*H*_3_-Ac), 1.14 (d, *J* = 6.6 Hz, 3H, C*H*_3_-6).

**General Procedure for Cross-Metathesis**

Compound **17** or **23** (1.00 mmol) was dissolved in dry CH_2_Cl_2_ (10 ml) under a nitrogen atmosphere. 1-Pentene or 1-heptene (0.6 ml) was added followed by addition of Grubbs 1^st^ generation catalyst (41 mg, 0.05 mmol), and the reaction mixture was refluxed overnight. The reaction was monitored by TLC and once complete, the mixture was concentrated. The crude product was purified by silica gel chromatography (toluene/EtOAc 8:1, v/v). Residual Grubb’s 1^st^ generation catalyst was removed from the compound by stirring it with activated charcoal in CH_2_Cl_2_ overnight, and filtering through Celite. Finally, the solvent was evaporated.

**(2*E*)-2-Hexen-1-yl 2,3,4-tri-*O*-acetyl-α-L-fucopyranoside** **(18)**

Following the general cross-metathesis procedure above, 100 mg (0.30 mmol) of **17** gave 94 mg (83%) of (2*E*)-2-hexen-1-yl 2,3,4-tri-*O*-acetyl-α-L-fucopyranoside **18** as a clear syrup. R_f_: 0.58 (toluene/EtOAc 4:1, v/v); ^1^H NMR (500 MHz, CDCl_3_) δ 5.75 – 5.67 (m, 1H, -OCH_2_CHC*H*-), 5.53 – 5.46 (m, 1H, -OCH_2_C*H*CH-), 5.37 (dd, *J* = 10.8, 3.4 Hz, 1H, H-3), 5.31 – 5.28 (m, 1H, H-4), 5.13 (dd, *J* = 10.8, 3.7 Hz, 1H, H-2), 5.09 (d, *J* = 3.7 Hz, 1H, H-1), 4.21 – 4.15 (m, 1H, H-5), 4.11 (ddd, *J* = 12.2, 5.7, 1.0 Hz, 1H, -OCH*H*CHCH-), 3.96 (ddd, *J* = 12.3, 6.9, 0.8 Hz, 1H, -OCH*H*CHCH-), 2.16 (s, 3H, C*H*_3_-Ac), 2.07 (s, 3H, C*H*_3_-Ac), 2.06 – 1.99 (m, 2H, -C*H*_2_CH_2_CH_3_), 1.98 (s, 3H, C*H*_3_-Ac), 1.41 (dd, *J* = 14.7, 7.3 Hz, 2H, -CH_2_C*H*_2_CH_3_), 1.14 (d, *J* = 6.6 Hz, 3H, C*H*_3_-6), 0.90 (t, *J* = 7.4 Hz, 3H, -CH_2_CH_2_C*H*_3_); ^13^C NMR (101 MHz, CDCl_3_) δ 170.60 - 169.99 (Cq-Ac), 135.58 (-OCH_2_CH*C*H-), 125.05 (-OCH_2_*C*HCH-), 94.88 (C-1), 71.25 (C-4), 68.50 (-O*C*H_2_CHCH-), 68.15 (C-2), 68.09 (C-3), 64.32 (C-5), 34.32 (-*C*H_2_CH_2_CH_3_), 22.11 (-CH_2_*C*H_2_CH_3_), 20.81 (*C*H_3_-Ac), 20.67 (*C*H_3_-Ac), 20.64 (*C*H_3_-Ac), 15.82 (C-6), 13.64 (-CH_2_CH_2_*C*H_3_); HRMS(ES^+^): calcd. for C_18_H_28_O_8_(Na^+^) requires 395.1682: found 395.1676; [α]_D_ = -136.6 (c 1.00, CHCl_3_)

**(2*E*)-2-Octen-1-yl 2,3,4-tri-*O*-acetyl-α-L-fucopyranoside (19)**

Following the general procedure, 100 mg (0.30 mmol) of **17** yielding 104 mg (86%) of (2*E*)-2-octen-1-yl 2,3,4-tri-*O*-acetyl-α-L-fucopyranoside **19** as a clear syrup. R_f_: 0.57 (toluene/EtOAc 4:1, v/v); ^1^H NMR (500 MHz, CDCl_3_) δ 5.74 – 5.67 (m, 1H, -OCH_2_CHC*H*CH_2_-), 5.52 – 5.45 (m, 1H, -OCH_2_C*H*CHCH_2_-), 5.37 (dd, *J* = 10.8, 3.4 Hz, 1H, H-3), 5.30 (d, *J* = 2.3 Hz, 1H, H-4), 5.13 (dd, *J* = 10.8, 3.7 Hz, 1H, H-2), 5.09 (d, *J* = 3.7 Hz, 1H, H-1), 4.21 – 4.16 (m, 1H, H-5), 4.09 (dd, *J* = 5.8, 0.9 Hz, 1H, -OCH*H*CHCHCH_2_-), 3.98 – 3.93 (m, 1H, -OCH*H*CHCHCH_2_-), 2.16 (s, 3H, C*H*_3_-Ac), 2.07 (s, 3H, C*H*_3_-Ac), 2.04 (dd, *J* = 14.5, 7.2 Hz, 2H, -OCH_2_CHCHC*H*_2_-), 1.98 (s, 3H, C*H*_3_-Ac), 1.44 – 1.34 (m, 2H, -C*H*_2_CH_2_CH_2_CH_3_), 1.29 (ttd, *J* = 12.6, 8.4, 4.3 Hz, 4H, -CH_2_C*H*_2_C*H*_2_CH_3_), 1.14 (d, *J* = 6.6 Hz, 3H, C*H*_3_-6), 0.89 (t, *J* = 7.0 Hz, 3H, -CH_2_CH_2_CH_2_C*H*_3_); ^13^C NMR (126 MHz, CDCl_3_) δ 170.63 - 170.02 (3C, Cq-Ac), 135.85 (-OCH_2_CH*C*HCH_2_-), 124.92 (-OCH_2_*C*HCHCH_2_-), 95.02 (C-1), 71.33 (C-4), 68.61 (-O*C*H_2_CHCHCH_2_-), 68.23 (C-2), 68.17 (C-3), 64.39 (C-5), 32.27 (-OCH_2_CHCH*C*H_2_-), 31.40 (-CH_2_CH_2_*C*H_2_CH_3_), 28.69 (-*C*H_2_CH_2_CH_2_CH_3_), 22.50 (-CH_2_*C*H_2_CH_2_CH_3_), 20.84 (*C*H_3_-Ac), 20.70 (*C*H_3_-Ac), 20.66 (*C*H_3_-Ac), 15.86 (C-6), 14.01 (-CH_2_CH_2_CH_2_*C*H_3_); HRMS (ES^+^): calcd. for C_20_H_32_O_8_(Na^+^) requires 423.1995: found 423.1999; [α]_D_ = -127.8 (c 1.00, CHCl_3_)

**General Procedure for Deacylation**

NaOMe was added to a solution of compound **18** or **19** or **24** (1.00 mmol) in dry MeOH (3 ml), until a pH of 12 was attained. The mixture was then stirred at RT for 3.5 h. Upon completion, the reaction was quenched with Dowex-50WX8 acid resin, filtered and concentrated. The crude product was purified by silica gel chromatography (EtOAc/isopropanol 99:1, v/v).

**(2*E*)-2-Hexen-1-yl α-L-fucopyranoside** **(20)**

Following the general deacylation procedure, 1.1 g (3.0 mmol) of **18** gave 701 mg (96%) of (2*E*)-2-Hexen-1-yl α-L-fucopyranoside **7** as a white solid. R_f_: 0.31 (EtOAc, 100%); ^1^H NMR (500 MHz, CD_3_OD) δ 5.67 – 5.60 (m, 1H, -OCH_2_CHC*H*-), 5.53 – 5.46 (m, 1H, -OCH_2_C*H*CH-), 4.69 (d, *J* = 3.0 Hz, 1H, H-1), 4.00 (ddd, *J* = 12.2, 5.8, 1.0 Hz, 1H, -OCH*H*CHCH-), 3.90 – 3.83 (m, 2H, H-5, -OCH*H*CHCH-), 3.62 (m, 2H, H-2, H-3), 3.56 – 3.54 (m, 1H, H-4), 1.94 (dd, *J* = 14.3, 7.3 Hz, 2H, -C*H*_2_CH_2_CH_3_-), 1.37 – 1.28 (m, 2H, -CH_2_C*H*_2_CH_3_), 1.11 (d, *J* = 6.6 Hz, 3H, C*H*_3_-6), 0.82 (t, *J* = 7.4 Hz, 3H, -CH_2_CH_2_C*H*_3_); ^13^C NMR (75 MHz, CDCl_3_) δ 135.76 (-OCH_2_CH*C*H-), 125.22 (-OCH_2_*C*HCH-), 97.15 (C-1), 71.76 (C-4), 71.63 (C-2), 69.55 (C-3), 68.58 (-O*C*H_2_CHCH-), 65.90 (C-5), 34.35 (-*C*H_2_ CH_2_CH_3_-), 22.15 (-CH_2_*C*H_2_CH_3_), 16.16 (C-6), 13.67 (-CH_2_CH_2_*C*H_3_); HRMS (ES^+^): calcd. for C_12_H_22_O_5_(Na^+^) requires 269.1365: found 269.1362; [α]_D_ = -106.4 (c 1.00, CH_3_OH).

**(2*E*)-2-Octen-1-yl α-L-fucopyranoside (21)**

Following the general procedure, 90 mg (0.23 mmol) of compound **19** yielded 61 mg (99%) of (2*E*)-2-octen-1-yl α-L-fucopyranoside **21** as a white solid. R_f_: 0.28 (EtOAc, 100%); ^1^H NMR (500 MHz, CD_3_OD) δ 5.63 (dd, *J* = 14.5, 7.6 Hz, 1H, -OCH_2_CHC*H*CH_2_-), 5.53 – 5.45 (m, 1H, -OCH_2_C*H*CHCH_2-_), 4.69 (d, *J* = 3.1 Hz, 1H, H-1), 4.00 (ddd, *J* = 12.1, 5.8, 1.0 Hz, 1H, -OCH*H*CHCHCH_2_-), 3.91 – 3.83 (m, 2H, H-5, -OCH*H*CHCHCH_2_-), 3.65 – 3.59 (m, 2H, H-2, H-3), 3.57 – 3.54 (m, 1H, H-4), 1.97 (dt, *J* = 20.7, 7.0 Hz, 2H, -OCH_2_CHCHC*H*_2_-), 1.30 (dd, *J* = 14.5, 7.2 Hz, 2H, -C*H*_2_CH_2_CH_2_CH_3_), 1.27 – 1.18 (m, 4H, -CH_2_C*H*_2_CH_2_CH_3_, -CH_2_CH_2_C*H*_2_CH_3_), 1.11 (d, *J* = 6.6 Hz, 3H, C*H*_3_-6), 0.81 (t, *J* = 7.0 Hz, 3H, -CH_2_CH_2_CH_2_C*H*_3_); ^13^C NMR (75 MHz, CDCl_3_) δ 136.04 (-OCH_2_CH*C*HCH_2_-), 125.00 (-OCH_2_*C*HCHCH_2_-), 97.17 (C-1), 71.75 (C-4), 71.64 (C-2), 69.54 (C-3), 68.60 (-O*C*H_2_CHCHCH_2_-), 65.90 (C-5), 32.26 (-OCH_2_CHCH*C*H_2_-), 31.39 (-CH_2_CH_2_*C*H_2_CH_3_), 28.68 (-*C*H_2_CH_2_CH_2_CH_3_), 22.50 (-CH_2_*C*H_2_CH_2_CH_3_), 16.17 (C-6), 14.03 (-CH_2_CH_2_CH_2_*C*H_3_).

**Hexyl α-L-fucopyranoside (22)**

Compound **20** (41 mg, 0.17 mmol) was dissolved in THF (0.8 ml). Pd/C 10mol% (14 mg, 0.13 mmol) was added. Under a hydrogen atmosphere the reaction was stirred overnight at RT. The reaction mixture was filtered through a series of filters (20μm, 10μm, 5μm) and concentrated. The crude product was purified by silica gel chromatography (CH_2_Cl_2_/MeOH 12:1, v/v) to yield **22** (35 mg, 85%) as a white solid. R_f_: 0.49 (CH_2_Cl_2_/MeOH 12:1, v/v); ^1^H NMR (500 MHz, CD_3_OD) δ 4.74 (d, *J* = 2.8 Hz, 1H, H-1), 3.95 (d, *J* = 6.6 Hz, 1H, H-5), 3.75 – 3.69 (m, 2H, H-2, H-3), 3.68 – 3.63 (m, 2H, H-4, -OC*H*_2_CH_2_CH_2_-), 3.45 (dt, *J* = 9.7, 6.4 Hz, 1H, -OC*H*_2_CH_2_CH_2_-), 1.69 – 1.58 (m, 2H, -OCH_2_C*H*_2_CH_2_-), 1.44 – 1.30 (m, 6H, -OCH_2_CH_2_C*H*_2_-, -C*H*_2_CH_2_CH_3_, -CH_2_C*H*_2_CH_3_), 1.21 (d, *J* = 6.6 Hz, 3H, C*H*_3_-6), 0.92 (dd, *J* = 8.5, 5.4 Hz, 3H, -CH_2_CH_2_C*H*_3_); ^13^C NMR (126 MHz, CDCl_3_) δ 98.19 (C-1), 71.80 (C-4), 71.59 (C-3), 69.60 (C-2), 68.39 (-O*C*H_2_CH_2_CH_2_-) , 65.82 (C-5), 31.54 (-OCH_2_CH_2_*C*H_2_-), 29.45 (-OCH_2_*C*H_2_CH_2_-), 25.80 (-*C*H_2_CH_2_CH_3_), 22.56 (-CH_2_*C*H_2_CH_3_), 16.18 (C-6), 14.01 (-CH_2_CH_2_*C*H_3_); HRMS (ES^+^): calcd. for C_12_H_24_O_5_(Na^+^) requires 271.1521: found 271.1516; [α]_D_ = -2.4 (c 1.20, CH_3_OH).

**FleA Inhibitor Synthesis**


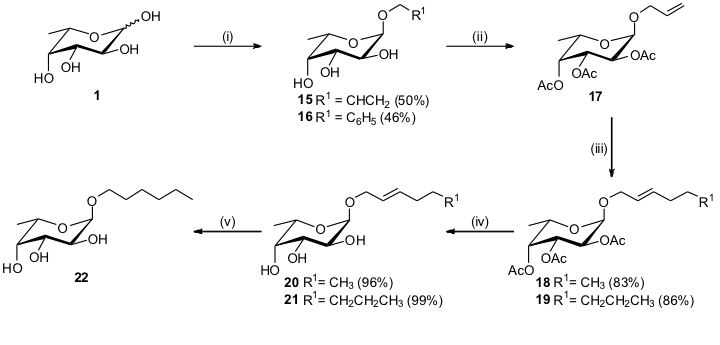


Scheme 4: (i) Allyl alcohol or benzyl alcohol, Dowex-50WX8, 70 °C; (ii) Ac_2_O, pyridine, RT, overnight, 91%; (iii) Grubbs 1^st^ generation catalyst, 1-pentene or 1-heptene, CH_2_Cl_2_, reflux, overnight; (iv) NaOMe, MeOH, RT; (v) H_2_, Pd/C 10mol%, THF, RT, overnight, 85%.

**Allyl 2,3,4,6-tetra-*O*-acetyl-D-galactoside (23)**

Synthesis as previously described [[8](#_ENREF_8)].

**(2*E*)-2-Hexen-1-yl 2,3,4,6-tetra-*O*-acetyl-α-D-galactopyranoside (24)**

Following the general cross-metathesis procedure above, 98 mg (0.25 mmol) of **23** yielded 90 mg (83%) of (2*E*)-2-hexen-1-yl 2,3,4,6-tetra-*O*-acetyl-α-D-galactopyranoside **24**. R_f_: 0.42 (toluene/EtOAc 4:1, v/v); ^1^H NMR (500 MHz, CDCl_3_) δ 5.75 – 5.68 (m, 1H, -OCH_2_CHC*H*-), 5.50 (dddd, *J* = 14.1, 8.5, 4.3, 2.9 Hz, 1H, -OCH_2_C*H*CH-), 5.46 (dd, *J* = 3.4, 1.3 Hz, 1H, H-4), 5.40 – 5.35 (m, 1H, H-3), 5.17 – 5.12 (m, 2H, H-1, H-2), 4.25 (td, *J* = 6.6, 1.3 Hz, 1H, H-5), 4.15 – 4.08 (m, 3H, -OCH*H*CHCH-, H-6a, H-6b), 3.97 (ddq, *J* = 12.1, 7.0, 0.9 Hz, 1H, -OCH*H*CHCH-), 2.14 (s, 3H, C*H*_3_-Ac), 2.08 (s, 3H, C*H*_3_-Ac), 2.05 (s, 3H, C*H*_3_-Ac), 2.05 – 2.01 (m, 2H, -C*H*_2_CH_2_CH_3_), 1.98 (s, 3H, C*H*_3_-Ac), 1.46 – 1.36 (m, 2H, -CH_2_C*H*_2_CH_3_), 0.91 (t, *J* = 7.4 Hz, 3H, -CH_2_CH_2_C*H*_3_); ^13^C NMR (126 MHz, CDCl_3_) δ 170.37 - 169.95 (Cq-Ac), 136.00 (-OCH_2_CH*C*H-), 124.77(-OCH_2_*C*H-), 94.83 (C-1), 68.60 (-O*C*H_2_CH-), 68.15 (C-4), 68.08 (C-2), 67.65 (C-3), 66.23 (C-5), 61.72 (C-6), 34.32 (-*C*H_2_CH_2_CH_3_), 22.11(-*C*H_2_CH_3_), 20.78 (*C*H_3_-Ac), 20.69 (*C*H_3_-Ac), 20.64 (*C*H_3_-Ac), 20.63 (*C*H_3_-Ac), 13.64 (-CH_2_*C*H_3_); [α]_D_ = +154.9 (c1.10, CHCl_3_).

**(2*E*)-2-Hexen-1-yl-α-D-galactopyranoside (25)**

Following the general procedure above, 61 mg (0.14 mmol) of **24** gave 33 mg (89%) of (2*E*)-2-hexen-1-yl-α-D-galactopyranoside **25** as a white solid. R_f_: 0.25 (CH_2_Cl_2_/MeOH 9:1, v/v); ^1^H NMR (300 MHz, CD_3_OD) δ 5.77 (m, 1H, -OCH_2_CHC*H*-), 5.68 – 5.56 (m, 1H, -OCH_2_C*H*CH-), 4.87 (d, *J* = 4.4 Hz, 1H, H-1), 4.18 (ddq, *J* = 11.6, 5.4, 1.1 Hz, 1H, -OCH*H*CHCH-), 4.00 (ddq, *J* = 11.9, 6.5, 0.9 Hz, 1H, -OCH*H*CHCH-), 3.90 (dd, *J* = 2.8, 1.3 Hz, 1H, H-4), 3.86 – 3.79 (m, 1H, H-5), 3.78 – 3.74 (m, 2H, H-2, H-3), 3.72 (dd, *J* = 6.1, 1.5 Hz, 2H, H-6a, H-6b), 2.13 – 2.00 (m, 2H, -C*H*_2_CH_2_CH_3_), 1.52 – 1.35 (m, 2H, -CH_2_C*H*_2_CH_3_), 0.94 (t, *J* = 7.4 Hz, 3H, -CH_2_CH_2_C*H*_3_); ^13^C NMR (126 MHz, D_2_O) δ 136.96 (-OCH_2_CH*C*H-), 124.68 (-OCH_2_*C*HCH-), 97.20 (C-1), 70.80 (C-5), 69.46 (C-2), 69.16 (C-4), 68.43 (-O*C*H_2_CHCH-), 68.10 (C-3), 61.05 (C-6), 33.68 (-*C*H_2_CH_2_CH_3_), 21.51 (-CH_2_*C*H_2_CH_3_), 12.90 (-CH_2_CH_2_*C*H_3_); HRMS (ES^+^): calcd. for C_12_H_22_O_6_(Na^+^) requires 285.1314: found 285.1313. [α]_D_ **=** +110.2 (c1.00, CH_3_OH).

Scheme 5: (i) Grubb’s 1^st^ generation catalyst, 1-pentene, CH_2_Cl_2_, reflux, overnight, 83%; (ii) NaOMe, MeOH, RT, 2 h, 89%.

**Aspergillus strains and culture**

All strains utilized or developed are listed in S2 Table. Unless noted, all *A. fumigatus* strains were propagated on solid glucose minimal media (GMM) at 37°C [[9](#_ENREF_9)]. *A. fumigatus* asexual spores were collected in water supplemented with 0.01% Tween 80, counted using a hemocytometer, and maintained at -80°C in 50% glycerol. Fixed spore suspensions were prepared as follows: 1 x 10^9^ spores were added to a 4% formaldehyde solution prepared in 1x PBS and allowed to incubate for 30 minutes at room temperature. Aldehydes were quenched by adding glycine to a final concentration of 100 mM and incubated at room temperature for an additional 10 minutes. Conidia were washed via centrifugation in Tween water three times and resuspended to a final concentration of 1 X 10^8^ spores/mL using Tween water. Killing of spores was confirmed by lack of growth on GMM media after three days.

**Generation of FleA mutant conidia**

Construction, isolation, and maintenance of fusion PCR products was carried out according to standard methods [[10](#_ENREF_10)]. Primers used are listed in Table S3. Genomic DNA was isolated from lyophilized hyphal tissue as previously described [[11](#_ENREF_11)]. The ORF of *A. fumigatus fleA* (Afu5g14740) was identified via the AspGD database (<http://www.aspergillusgenome.org>).

Nuclear-tagged GFP *A. fumigatus* strains were created by transforming *A. fumigatus* with a plasmid termed pJMP51 containing histone H2A N-terminally fused to GFP driven by the constitutive *gpdA* promoter (*gpdA(p)*::GFP::H2A) and harboring the *A. fumigatus pyrG* gene for selection. Briefly, pJMP51 was created from pSK505 [[12](#_ENREF_12)] which already contained the *gpdA(p)*::GFP::H2A cassette by enzymatic digestion with *ClaI*. A *ClaI-ClaI* fragment of *Aspergillus fumigatus pyrG* was then amplified with the primer pair “JP AfpyrG ClaI For/Rev” and sub-cloned into the *ClaI* site to create pJMP51. pJMP51 was used to transform AF293.1 and AF293.6 to construct TJMP131.5 (*GFP::H2A*) and TGJF5.3 (*GFP::H2A, argB1*) respectively. Integration was confirmed by Southern blot (data not shown) and nuclear GFP signal confirmed via microscopy. We then generated a gene disruption cassette for *fleA* using published protocols [[13](#_ENREF_13)] and transformed the cassette into TGJF5.3 to create TGJF6.7 (*GFP::H2A, ∆fleA*). The *fleA* disruption cassette was constructed by fusion of a 2kb region upstream and downstream of the *fleA* open reading frame amplified using “GF FleA del F (P1)” with “GF FleA del R (P3)” and “GF FleA del F (P6)” with “GF FleA del P8 R”, respectively. The *A. fumigatus argB* gene was amplified via genomic DNA using “GF A. fumi argB F (P4)” with “GF A. fumi argB R (P5)” and fused to the flanking *fleA* PCR fragments using “GF FleA del F (P2)” and “GF FleA del P7 R” primers via fusion PCR [[13](#_ENREF_13)]. Deletion of *fleA* was confirmed by Southern and northern blot (Figure 2B,C).

To identify FleA localization, the native *fleA* locus was tagged with RFP through double homologous recombination using the *A. fumigatus argB* gene as a marker. For RFP tagging, the auxotrophic marker, *pyrG,* was replaced with *A. fumigatus argB* within pXDRFP4 [[14](#_ENREF_14)] via QuickChange site-directed mutagenesis [[15](#_ENREF_15)] using the primer pair “GF argB QC F (pGJF7)” and “GF argB QC R (pGJF6-7)”, yielding the plasmid, pGJF7.2. A gene disruption cassette was developed as described for deletion of *fleA.* Briefly, a 2kb region upstream and downstream of the *fleA* stop codon was amplified using “GF FleA Native Tag P1” with “GF FleA Native Tag P3” and “GF FleA Native Tag P6” with “GF FleA del P8 R”, respectively. An *rfp/argB* cassette was amplified from pGJF7.2 using “GF/JP GFP/RFP F” with “GF/JP GFP/RFP R” and fused to the flanking *fleA* PCR fragments using “GF FleA Native Tag P2” and “GF FleA del P7” primers via fusion PCR [[13](#_ENREF_13)]. TGJF5.3 was then transformed using the cassette yielding the prototrophic *fleA:RFP* strain, TGJF7.11. FleA tagging was confirmed microscopically and using Southern and northern blots (Figure 2 D,E).

The *fleA* deletion mutants in *A. flavus* were made by targeted integration of a deletion cassette via transformation. To construct the *fleA* deletion cassette in *A. flavus*, a 1.0 - 1.5 kb region flanking the *fleA* open reading frame was amplified from gDNA (5’ Flank: “FY AFLA FleA 5’ FOR” with “FY AFLA FleA 5’ REV”, 3’ Flank: “FY AFLA FleA 3’ FOR” with “FY AFLA FleA 3’ REV”). The flanking fragments were fused to the selectable auxotrophic marker (*A. fumigatus* *pyrG,* amplified with primer pair “KS Afu pyrG FOR” and “KS Afu pyrG REV”) using a double-joint fusion PCR approach (“FY AFLA FleA Nest FOR” with “FY AFLA FleA Nest REV”) as described above. The deletion construct was transformed into parental strain CA14*∆ku70∆pyrG* [[16](#_ENREF_16)] to create strains TFYL62.1-62.3. Single integration of the deletion cassette was verified via Southern analysis using two distinct restriction digests (Fig. S2).

**Immunofluorescence of *Aspergillus* conidia**

For imaging of spores, *A. fumigatus* strains were cultured on GMM at 37°C for 3 days, and spores harvested in 0.1% Tween 20. 2μl of spore suspension was plated on a pre-cleaned glass slide with 15 µL of H_2_O spotted in the center of the slide, a coverslip was added, and sealed with nail polish. Images were taken using a Nikon T*i* inverted microscope equipped with a Nikon Plan Apo VC 60x/1.40 Oil DIC/∞/0.17 WD objective and a Nikon Intensilight C-HGFIE light source using the Nikon NIS-Elements Advanced Research V3.22 software package. Microscope settings were kept identical for all images. Time-course microscopy was carried out over 27 hours at 37°C to monitor FleA production on hyphae, resting conidia, and swollen conidia using the Nikon NIS Elements-AR software package (v.4.3). The average fluorescent intensity at each developmental state (resting conidia, swollen conidia, and hyphae) of untagged FleA (TJMP131.5 or wild type) was subtracted from the mean fluorescent intensity value of two different transformants (TGJF7.11 and TGJF7.15) expressing RFP-tagged versions of FleA. The adjusted mean fluorescence was then standardized to area. The adjusted fluorescence/area of the different developmental states were then compared using the Student’s T-test (n≥13).

**Western blot of *A.fumigatus* extracts and supernatant.**

Resting and swollen *A. fumigatus* conidia and hyphae were isolated from WT and *ΔfleA* cultures. Resting conidia grown on solid GMM were immediately collected in liquid GMM, enumerated, and diluted to a final concentration of 8.3x10^6^ spores/mL. Six milliliters of spore suspension (5x10^7^ spores) was separated from supernatant via centrifugation at 13,000 RPM for 5 minutes and resting conidia supernatant immediately frozen. For swollen conidia, 5X10^7^ spores were inoculated in 50 mL liquid GMM (1X10^6^ spores/mL) and grown 6 hours at 37°C, after which swollen spore tissue was separated from supernatant as described above. Hyphal tissue and supernatant was collected from identical growth conditions after 24 hours at 37°C. Hyphal tissue was lyophilized and ground to a fine powder. Tissue from resting and swollen conidia and 100 uL of ground hyphal powder were resuspended in 1 mL 50mM Tris/HCl pH 7.4, 50 mM EDTA, 2% SDS, and 40 mM β-Mercaptoethanol and homogenized using sterile 0.5 mm beads and beat for 5 minutes. After, extracts were boiled for 1 hour and centrifuged to remove insoluble material. Protein concentrations were determined by BCA assay and 15 μg of protein was loaded into each well of a 4-12% BOLT SDS PAGE gel (Life Technologies, Grand Island, NY) and electrophoresed under reducing conditions. The gel was then blotted onto nitrocellulose, blocked with 5% non fat milk for 2 hours and stained with an anti-FleA rabbit polyclonal antibody at 1μg/ml [[1](#_ENREF_1)] and donkey anti-rabbit HRP (Jackson Immunoresearch, West Grove, PA) prior to chemiluminescent detection. Culture supernatants were filtered through a 0.2 μM filter and concentrated 10x in a 0.5 ml 3 kDa, MWCO Amicon Ultra (EMD Millipore, Billirica, MA) before being run as described above.

**Mucin conidia binding assay**

8 well glass chamber slides (Labtek, Scotts Valley, CA) were coated with 20 μg/ml purified human mucin in MilliQ dH_2_O overnight at 37^o^C until material was dried onto surface. The slide was then blocked in PBS + 1% BSA for 1 hour at room temperature. Fixed conidia suspensions were centrifuged at 6000 x g for 5 minutes to pellet and resuspended in PBS + 1% BSA in the presence or absence of 10 mM (2E)-hexenyl α-L-fucopyranoside (2EHex) or 100 mM fucose. 2x10^7^ conidia were added per well and incubated for 4 hours at room temperature. Unbound conidia were removed by washing in PBS+ BSA, the slides were mounted in Prolong Gold anti-fade reagent (Life Technologies, Grand Island, NY) and allowed to cure for 24 hours prior to sealing. Images were acquired using an FV10i confocal microscope (Olympus, Center Valley, PA) using the multipoint Z-stack mode to acquire 9 fields per well with 3 wells imaged per condition per experiment. Each Z-stack image was compressed into a single plane of focus and conidia were counted using NIH Image J with the ITCN plugin. Each experiment was repeated at least 3 times. *A. flavus*-mucin interactions were investigated as described above with one exception. These conidia lack GFP so were stained with Calcofluor white for 5 minutes to allow imaging prior to adding to mucin-coated slides.

**Cell culture**

RAW 264.7 cells were obtained from the UCSF cell culture facility and maintained in DMEM + 10% fetal bovine serum + 1% penicillin/streptomycin. Cells were seeded and grown on 8 well chamber slides (Labtek, Scotts Valley, CA) overnight prior to use in assays. Primary alveolar macrophages were obtained from bronchoalveolar lavage (BAL) taken during research bronchoscopy. BAL was centrifuged at 450 x g for 10 minutes and cells were washed with PBS prior to plating on poly-L-lysine coated 8 well chamber slides in RPMI 1640+ 10% fetal bovine serum + 1% penicillin/streptomycin + 0.5μg/ ml amphotericin B. Cells were washed after 2 hours of adherence and cultured overnight prior to use in experiments. All cell culture reagents were purchased from Life Technologies, Grand Island, NY.

**Flow cytometry analysis of macrophages**

Recombinant FleA was tagged with Alexa-488 using the microscale labeling kit according to manufacturers instructions (Life Technologies, Grand Island NY). RAW264.7 macrophages were harvested by scraping or primary human lung macrophages were freshly isolated from bronchoalveolar lavage from research bronchoscopy. 5x10^5^ cells per sample were washed with PBS and incubated with 20 μg/ml recombinant FleA-488 in PBS + 0.25% BSA + 0.02% sodium azide (PBA) on ice, in the dark for 1 hour. Controls included unstained cells and FleA in the presence of 100 mM fucose. Cells were washed three times in PBA and fixed in 4% paraformaldehyde for 20 minutes at room temperature. Cells were analyzed using a FACScalibur (Beckton Dickenson, San Jose, CA) and Flow Jo software (Treestar, Ashland, OR).

**Confocal phagocytosis assay**

RAW 264.7 cells were plated at 5x10^4^ per well of an 8 well chamber slide (Labtek, Scotts Valley, CA) and allowed to grow overnight in culture media. 5x10^6^ condia from either the PFA fixed WT or *ΔfleA* strain were added per well in a 1:10 dilution of growth media in the presence or absence of 10 mM (2E)-hexenyl α-L-fucopyranoside or 500 mM fucose and incubated at 37^o^C for 1 hour. Wells were washed gently with warm media and incubated for a further 2 hours at 37^o^C for complete uptake. Cells were then incubated with a 7.5 μg/ml solution of CellMask Deep Red plasma membrane stain (Life Technologies, Grand Island, NY) for 5 minutes at 37^o^C, prior to staining with calcofluor white for 1 minute at room temperature (Sigma, St Louis, MO), washing with PBS and mounting with Fluoromount-G (Southern Biotech, Birmingham, AL). Z-stack images were acquired using an FV10i confocal microscope (Olympus, Center Valley, PA). Each Z-stack image was compressed into a single plane of focus and conidia were manually counted using NIH Image J with the cell counter plugin. Internalized conidia were counted as conidia within the boundary of the cell that were not stained with Calcofluor white. Calcofluor white stained cells were excluded from the count. For primary human alveolar macrophages, cells were plated at 5x10^5^ per well on a poly-L-lysine coated 8 well chamber slide (Labtek, Scotts Valley, CA) and allowed to grow overnight in culture media. 1.5x10^6^ conidia from either the PFA fixed WT or *ΔfleA* strains were added per well in a 1:10 dilution of growth media in the presence or absence of 10 mM (2E)-hexenyl α-L-fucopyranoside or 500 mM fucose, incubated at 37^o^C for 30 minutes then washed, stained and mounted as described above.

**Flow cytometry analysis of *A. fumigatus* conidia.**

Recombinant Dectin-1 was purchased from R&D Systems (Minneapolis, MN) and biotinylated using the EZ-link sulfo NHS biotin kit (Thermo Fisher Waltham, MA) according to manufacturers recommendations. 1x10^7^ WT or *ΔfleA* conidia were centrifuged and resuspended in a solution of 20 μg/ml biotinylated Dectin-1 in PBS for 1 hour at 4^o^C. Conidia were washed in PBS and the Dectin-1 detected using Streptavidin-PE (Biolegend, San Diego, CA) in PBS at 20 μg/ml for a further 1 hour at 4^o^C. Conidia were washed in PBS then fixed in 4% paraformaldehyde prior to analysis on a Becton Dickenson FACSCalibur and FlowJo software (TreeStar, Ashland, OR).

**Phagocytosis of FleA-coated particles.**

Yellow-green 1μM sulfate treated FluoSpheres (Life Technologies, Grand Island, NY) were coated with 50μg/ml recombinant FleA in H_2_O and incubated for 2 hours at room temperature. Beads were washed 3 times to remove unattached protein and resuspended in 1:10 dilution of RAW264.7 growth media in the presence or absence of 500 mM fucose and incubated for 30 minutes with agitation. FluoSpheres were added to RAW264.7 cells at a ratio of 10:1 and incubated with agitation for 2 hours at 37^o^C prior to extensive washing with DMEM to remove unattached FluoSpheres from the cell surface. Cells were fixed in 4% paraformaldehyde prior to analysis on a Becton Dickenson FACSCalibur and FlowJo software (TreeStar, Ashland, OR).

**Mice**

Male C57BL/6 mice were purchased from Charles River Laboratories (Wilmington, Massachusetts, MA) and housed in a specific pathogen free facility at the University of California, San Francisco (UCSF). Animal experiments followed protocols approved by the UCSF Institutional Animal Care and Use Committee.

**Mouse model of Aspergillus infection**

Eight to ten week old C57BL/6 mice (Charles River Laboratories, Hollister, CA), were anaesthetized with isoflurane and infected intranasally with 5x10^7^ of either WT or *ΔfleA* conidia. Mice were sacrificed 3 days post infection. For the first experiment, 5 mice were treated per group and analyzed by flow cytometry for lung inflammation with histology, qPCR and hemoglobin measurements made for assessment of fungal burden and invasive disease. For the second experiment 10 mice were treated per group and analysis focused on fungal burden, hemoglobin measurements and histological outcomes. The left lung lobe was snap frozen in liquid nitrogen for RNA analysis. The remaining lung lobes were either fixed for histology analysis or used for flow cytometry analysis. For histology, lung lobes were fixed in 4% PFA and subsequently embedded into paraffin, sectioned and stained for H&E or Grocott’s Methanamine Silver (GMS) stain by the UCSF Pathology core. Snap frozen lung was homogenized in 1.2 ml of RLT (Qiagen, Valencia, CA) containing 0.14 M β-ME using Tissumizer (Tekmar, Cincinnati, Ohio). From that lysate, 600 μl was spun at 20,000 x g for 3 minutes. RNA was purified from the clarified lysate using the RNeasy mini kit with on-column DNase digestion (Qiagen, Valencia, CA) according to manufacturer’s protocol. 1 μg of RNA was used to make cDNA with the iScript cDNA kit (Biorad, Hercules, CA) following manufacturer’s protocols. Real-time qPCR was performed using 2 µl of cDNA in a 25 µl reaction to measure the relative levels of *Aspergillus* 18S RNA. The HPRT gene was used as a loading control to normalize the amount of cDNA input in the PCR reaction. All primer sequences used are shown in the S3 Table.

Bronchoalveolar lavage (BAL) was performed with 1 ml of ice cold PBS + 2% FBS. BAL was centrifuged at 1500 rpm for 5 minutes at 4^o^C. The supernatant was removed and 10 μl was used to measure the hemoglobin (Hb) concentration using a Plasma / Low Hb Photometer (HemoCue AB, Angelholm, Sweden). Two million BAL cells were transferred into V-bottomed 96 well plates for flow cytometric staining and analysis. Cells were spun down and resuspended in 25 µl/well FACS buffer (PBS with 2% FBS, 1mM EDTA and 0.1% sodium azide) containing Fc receptor (CD16/32) blocking antibody (clone 2.4G2). After 15 min incubation on ice, fluorescent-conjugated antibodies diluted in 25 µl/well FACS buffer were added to the cells for 45 mins on ice. The cells were washed twice with 200 µl/well FACS buffer and resuspended in 100 µl/well FACS buffer containing propidium iodide (0.5 µg/ml) for live/dead discrimination. Samples were acquired on an LSRFortessa cell analyzer (BD Biosciences) in the DERC Flow Cytometry and Cell Sorting Core Facility at UCSF. Data analysis was done using FlowJo software (TreeStar, Ashland, OR). Antibodies used for flow cytometric staining were: CD11c (clone HL3), F4/80 (clone CI:A3-1), CD11b (clone M1/70), MHCII ( clone M5/114.15.2), Ly6G (clone 1A8), Ly6C (HK1.4), NK1.1 (PK136 ), TCRβ (H57-597), B220, CD4 and CD8. Antibodies were purchased from BD Biosciences, eBiosciences or Biolegend.

**Quantification of conidia germination.**

GMS stained slides of mouse lung infected with WT or *ΔfleA* conidia (n=5 per group) were examined at 20X using an integrated microscope (Olympus, Albertslund, Denmark), video camera (JVC Digital Color; JVC, Tatstrup, Denmark), automated microscope stage, and computer (Dell Optiplex GS270 PC running Computer-Assisted Stereology Toolbox (CAST) software; Olympus, Albertslund, Denmark) [[17](#_ENREF_17)]. For each slide, the software systematically selected at least 20 fields, and the number of conidia and the number of germlings were counted in each field.

- Number of conidia: The number of germlings was expressed as the number per lung surface area (μM^2^). The surface area of lung tissue was quantified using the point-counting and line-segment grid tools available in the CAST software.
- Percentage of germinating conidia: The percentage of germinating conidia was calculated as the number of germinating conidia divided by the sum of non-germinating and germinating conidia (x100).

**Statistical methods**

Data analyses were performed GraphPad Prism version 6 (GraphPad, San Diego, CA). ANOVA was used for three-group comparisons followed by pairwise analyses with the Tukey multiple comparisons test when appropriate. Two group comparisons were made using Students t-test or for non parametric analyses, a ranked Mann-Whitney test.

**Supporting Information References**

1. Houser J, Komarek J, Kostlanova N, Cioci G, Varrot A, Kerr SC, et al. A soluble fucose-specific lectin from *Aspergillus fumigatus* conidia--structure, specificity and possible role in fungal pathogenicity. PLoS One. 2013;8(12):e83077. doi: 10.1371/journal.pone.0083077. PubMed PMID: 24340081; PubMed Central PMCID: PMC3858362.

2. Kostlanova N, Mitchell EP, Lortat-Jacob H, Oscarson S, Lahmann M, Gilboa-Garber N, et al. The fucose-binding lectin from *Ralstonia solanacearum*. A new type of beta-propeller architecture formed by oligomerization and interacting with fucoside, fucosyllactose, and plant xyloglucan. J Biol Chem. 2005;280(30):27839-49. doi: 10.1074/jbc.M505184200. PubMed PMID: 15923179.

3. Katoh K, Misawa K, Kuma K, Miyata T. MAFFT: a novel method for rapid multiple sequence alignment based on fast Fourier transform. Nucleic Acids Research. 2002;30(14):3059-66. PubMed PMID: 12136088; PubMed Central PMCID: PMC135756.

4. Larsson A. AliView: a fast and lightweight alignment viewer and editor for large datasets. Bioinformatics. 2014;30(22):3276-8. doi: 10.1093/bioinformatics/btu531. PubMed PMID: 25095880; PubMed Central PMCID: PMC4221126.

5. Price MN, Dehal PS, Arkin AP. FastTree: computing large minimum evolution trees with profiles instead of a distance matrix. Molecular Biology and Evolution. 2009;26(7):1641-50. doi: 10.1093/molbev/msp077. PubMed PMID: 19377059; PubMed Central PMCID: PMC2693737.

6. Peters MC, Mekonnen ZK, Yuan S, Bhakta NR, Woodruff PG, Fahy JV. Measures of gene expression in sputum cells can identify TH2-high and TH2-low subtypes of asthma. J Allergy Clin Immunol. 2014;133(2):388-94. doi: 10.1016/j.jaci.2013.07.036. PubMed PMID: 24075231; PubMed Central PMCID: PMC3981552.

7. Royle L, Matthews E, Corfield A, Berry M, Rudd PM, Dwek RA, et al. Glycan structures of ocular surface mucins in man, rabbit and dog display species differences. Glycoconj J. 2008;25(8):763-73. doi: 10.1007/s10719-008-9136-6. PubMed PMID: 18465222.

8. Helferich B, Kashelikar DV. A new method for the synthesis of α-glycosides. Chemische Berichte. 1957;90:2094-6.

9. Shimizu K, Keller NP. Genetic involvement of a cAMP-dependent protein kinase in a G protein signaling pathway regulating morphological and chemical transitions in *Aspergillus nidulans*. Genetics. 2001;157(2):591-600. PubMed PMID: 11156981; PubMed Central PMCID: PMC1461531.

10. Sambrook J, Fritsch E. F., Maniatis T. Molecular cloning : a laboratory manual. Cold Spring Harbor, N.Y.: Cold Spring Harbor Laboratory; 1989.

11. Innis MA, Gelfand DH, Sninsky JJ, White TJ, editors. PCR Protocols: a guide to methods and applications: Academic Press; 1990.

12. Szewczyk E, Krappmann S. Conserved regulators of mating are essential for *Aspergillus fumigatus* cleistothecium formation. Eukaryotic Cell. 2010;9(5):774-83. doi: 10.1128/EC.00375-09. PubMed PMID: 20348388; PubMed Central PMCID: PMC2863953.

13. Szewczyk E, Nayak T, Oakley CE, Edgerton H, Xiong Y, Taheri-Talesh N, et al. Fusion PCR and gene targeting in *Aspergillus nidulans*. Nature Protocols. 2006;1(6):3111-20. doi: 10.1038/nprot.2006.405. PubMed PMID: 17406574.

14. Yang L, Ukil L, Osmani A, Nahm F, Davies J, De Souza CP, et al. Rapid production of gene replacement constructs and generation of a green fluorescent protein-tagged centromeric marker in *Aspergillus nidulans*. Eukaryotic Cell. 2004;3(5):1359-62. doi: 10.1128/EC.3.5.1359-1362.2004. PubMed PMID: 15470263; PubMed Central PMCID: PMC522605.

15. Bok JW, Keller NP. Fast and easy method for construction of plasmid vectors using modified quick-change mutagenesis. Methods Mol Biol. 2012;944:163-74. doi: 10.1007/978-1-62703-122-6_11. PubMed PMID: 23065615; PubMed Central PMCID: PMC3692276.

16. Chang PK, Scharfenstein LL, Wei Q, Bhatnagar D. Development and refinement of a high-efficiency gene-targeting system for *Aspergillus flavus*. Journal of Microbiological Methods. 2010;81(3):240-6. doi: 10.1016/j.mimet.2010.03.010. PubMed PMID: 20298723.

17. Woodruff PG, Boushey HA, Dolganov GM, Barker CS, Yang YH, Donnelly S, et al. Genome-wide profiling identifies epithelial cell genes associated with asthma and with treatment response to corticosteroids. Proc Natl Acad Sci USA. 2007;104(40):15858-63. doi: 10.1073/pnas.0707413104. PubMed PMID: 17898169; PubMed Central PMCID: PMCPMC2000427.
